# Full activation pattern mapping by simultaneous deep brain stimulation and fMRI with graphene fiber electrodes

**DOI:** 10.1038/s41467-020-15570-9

**Published:** 2020-04-14

**Authors:** Siyuan Zhao, Gen Li, Chuanjun Tong, Wenjing Chen, Puxin Wang, Jiankun Dai, Xuefeng Fu, Zheng Xu, Xiaojun Liu, Linlin Lu, Zhifeng Liang, Xiaojie Duan

**Affiliations:** 1 Department of Biomedical Engineering, College of Engineering, Peking University, Beijing, 100871 China; 2 Academy for Advanced Interdisciplinary Studies, Peking University, Beijing, 100871 China; 3 Institute of Neuroscience, Chinese Academy of Sciences, CAS Center for Excellence in Brain Sciences and Intelligence Technology, Key Laboratory of Primate Neurobiology, Chinese Academy of Sciences, Shanghai, 200031 China; 4 School of Biomedical Engineering, Guangdong Provincial Key Laboratory of Medical Image Processing, Key Laboratory of Mental Health of the Ministry of Education, Southern Medical University, Guangzhou, 510515 China

## Abstract

Simultaneous deep brain stimulation (DBS) and functional magnetic resonance imaging (fMRI) constitutes a powerful tool for elucidating brain functional connectivity, and exploring neuromodulatory mechanisms of DBS therapies. Previous DBS-fMRI studies could not provide full activation pattern maps due to poor MRI compatibility of the DBS electrodes, which caused obstruction of large brain areas on MRI scans. Here, we fabricate graphene fiber (GF) electrodes with high charge-injection-capacity and little-to-no MRI artifact at 9.4T. DBS-fMRI with GF electrodes at the subthalamic nucleus (STN) in Parkinsonian rats reveal robust blood-oxygenation-level-dependent responses along the basal ganglia-thalamocortical network in a frequency-dependent manner, with responses from some regions not previously detectable. This full map indicates that STN-DBS modulates both motor and non-motor pathways, possibly through orthodromic and antidromic signal propagation. With the capability for full, unbiased activation pattern mapping, DBS-fMRI using GF electrodes can provide important insights into DBS therapeutic mechanisms in various neurological disorders.

## Abstract (web summary)

Combination of fMRI and deep brain stimulation (DBS) allows for large-scale mapping of brain responses to DBS. Here the authors develop highly MRI compatible graphene fiber electrodes for full brain activation pattern mapping under DBS in Parkinsonian rats.

## Introduction

The electrical stimulation of neural tissues forms the basis of current and emerging neural prostheses and therapies, including deep brain stimulation (DBS) for movement disorders, cochlear implants for deafness, retinal and cortical implants for blindness, spinal cord stimulation for chronic pain, limb stimulation for stroke and spinal cord injury, and vagus nerve stimulation for epilepsy and depression^1–5^. The therapeutic mechanisms and neuromodulatory effects of electrical stimulation, such as DBS, remain poorly understood, despite its widespread utilization^6,7^. Electrical stimulation of brain tissue may evoke various responses at both the local and global levels, but a comprehensive study of these effects is challenging to undertake using the electrophysiology techniques due to limited data sampling from predefined anatomical brain regions. Functional magnetic resonance imaging (fMRI) represents a powerful tool for mapping brain activity on a whole-brain scale. Simultaneous DBS and fMRI (DBS–fMRI) thus could provide us with valuable insights into brain function and connectivity patterns, as well as modulatory effects and therapeutic mechanisms of functional electrical stimulation in various neurological disorders^8–10^.

A major obstacle to combine electrical brain stimulation and fMRI is that many metal electrodes elicit strong magnetic field interference and produce significant artifacts, which obstructs functional and structural mapping of a large volume of brain tissues surrounding the electrodes^11–13^. The main cause for this magnetic field distortion is the mismatch of magnetic susceptibility between the electrodes and water/tissues. The image artifacts or blind spots around the electrodes are particularly severe in fMRI, as it is more susceptible to such effect^14^. This artifact may not affect the identification of long-range responses in brain areas far from the implanted electrode, but local responses at the stimulation site, as well as activation of brain nuclei close to the implanted electrode tracks, will be obstructed, giving rise to incomplete and biased activation pattern mapping^11^. In addition to materials, the electrode size is another important factor to determine MRI artifact size and comprehensiveness of activation pattern mapping. Electrode materials with high charge-injection-capacity and stability are highly desirable to decrease MRI artifact size, as well as to improve stimulation resolution, and to elicit effective and chronically stable brain responses with minimal tissue damage.

In this work, we report on a full and unbiased activation pattern mapping by DBS–fMRI with graphene fiber (GF) microelectrodes in a Parkinson’s disease (PD) rat model. Graphene films have been utilized in various kinds of neural electrodes^15–17^, due to the unique electrical and optical properties. Here, we show that microelectrodes made from GFs exhibit the combined advantages of high charge-injection-capacity, stimulation stability, and MRI compatibility, which were not achievable by other electrodes. High-frequency DBS targeted at the subthalamic nucleus (STN) with GF electrodes effectively alleviates motor deficits in rats with PD. Moreover, the little-to-no artifact of the GF electrodes in various anatomical and functional MRI images makes all brain regions accessible by fMRI mapping under simultaneous DBS. STN–DBS in PD rats with GF microelectrodes evokes robust blood-oxygenation-level-dependent (BOLD) responses in multiple cortical and subcortical regions along the basal ganglia–thalamocortical network in a frequency-dependent manner. The BOLD responses of some of these regions were not previously detectable with traditional metal electrodes due to their large artifact^11^. The activation pattern indicates that STN–DBS modulates both motor and non-motor pathways, possibly through orthodromic and antidromic signal propagation. We believe that the DBS–fMRI studies with GF electrodes can serve as a powerful platform for translational research investigating the therapeutic mechanisms and modulatory effects of DBS.

## Results

### GF microelectrode technology

**Fig. 1:**
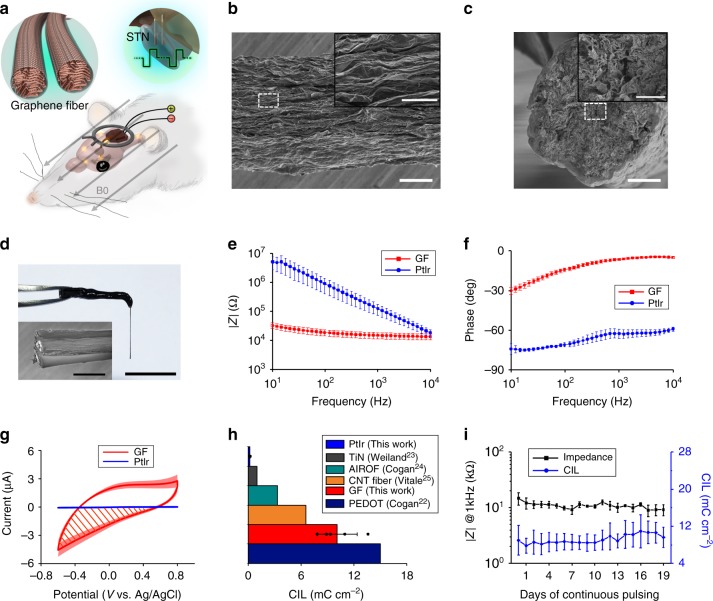
GF electrodes characterization. **a** A schematic drawing of the DBS–fMRI study using GF bipolar microelectrodes. **b** A representative SEM image of the axial external surface of a GF fiber. Inset, magnified image of the region in the dashed box. Scale bar, 20 μm; inset, 5 μm. **c** A typical SEM image of the exposed cross section acting as the active stimulating site of a GF electrode. Inset, magnified image of the region in the dashed box. Scale bar, 20 μm; inset, 5 μm. Experiments were repeated five times (for **b**) and three times (for **c**) with similar results. **d** The picture of a GF bipolar microelectrode assembly. Inset, SEM image of the GF bipolar microelectrode tip, showing two GFs (bright core) with each one insulated with Parylene-C film (dark shell). Scale bar, 1 cm; inset, 100 μm. **e**, **f** Impedance modulus and phase of GF and PtIr microelectrodes. **g** Cyclic voltammetry of GF and PtIr electrodes. The time integral of the negative current shown by the shadow region represents the CSC_c_. **h** CIL of different electrode materials. “AIROF” means activated iridium oxide film. **i** Stability of GF microelectrodes under continuous overcurrent pulsing at 1 mA current amplitude and 130 Hz frequency (see “Methods” for detailed pulsing parameters). Data represented as mean ± SD in **e**–**i** (*n* = 5 electrodes). Source data are provided as a Source Data file.

GFs were prepared through a dimension-confined hydrothermal process from aqueous graphite oxide (GO) suspensions^18^. Briefly, a glass pipeline was filled with an aqueous GO suspension. After being baked at 230 °C for 2 h with the pipeline sealed, a GF matching the pipe geometry was produced. The as-prepared GFs had a loose structure, with the graphene sheets randomly oriented. After being air-dried, the GO sheets became densely stacked and aligned parallel to the fiber’s main axis, which causes a shrinkage of the fiber diameter and provides GFs with high electrical conductivity, as well as excellent mechanical strength and robustness, which is superior to carbon fibers (CFs), known to have poor fracture resistance and is inconvenient to use^18–20^. The fiber diameter was determined by the pipeline dimension and the GO concentration^18^. A diameter of ~75 μm was used throughout this work. The electrodes made from GFs of this diameter are mechanically strong enough for self-supported implantation into the brain. A typical scanning electron microscopy (SEM) image of a GF shows a porous structure with easily defined individual graphene sheets aligned along the axis (Fig. 1b). The Raman spectrum of the fibers revealed characteristic G and D peaks of the GO (Supplementary Fig. 1)^21^. Electrode fabrication started with insulating individual GFs with Parylene-C film of ∼5 μm thickness. A pair of insulated GFs were aligned in parallel and pasted together with glue. After soldering one end of the two GFs onto a custom-made MRI-compatible connector made of high-purity copper used to interface with the stimulation pulse generator, the GFs were mechanically cut to expose the cross sections as electrically active sites, completing the fabrication of a bipolar GF-stimulating microelectrode. The high porosity and roughness of the exposed cross sections of the GF microelectrodes (Fig. 1c) resulted in a large surface area, which is advantageous for achieving high charge-injection-capacity and low impedance^22^. In addition, due to their high mechanical robustness, the GFs can be bent 90° to accommodate the MRI surface coil receiver during MRI scans.

The picture of a typical GF bipolar microelectrode is shown in Fig. 1d. Electrochemical impedance spectroscopy (EIS) measurements resulted in impedance values of 15.1 ± 3.67 kΩ at 1 kHz (mean ± SD, *n* = 5) for GF microelectrodes, which is approximately eight times lower than that of PtIr electrodes of the same diameter (126 ± 53.8 kΩ at 1 kHz, mean ± SD, *n* = 5) (Fig. 1e). The cyclic voltammogram (CV) of GF electrodes exhibited a nearly rectangular shape with no redox peaks observed (Fig. 1g). This suggested that the electrochemical interaction at the GF electrode–electrolyte interface is controlled by capacitive rather than Faradic process. The more resistive phase angle of the GF electrodes compared with PtIr electrodes (Fig. 1f) indicates the reduced imaginary component of impedance as a result of the large electrode surface area and double-layer capacitance^22^.

The cathodal charge-storage-capacity (CSC_c_) was calculated over a potential range of −0.6 to 0.8 V (shaded region in Fig. 1g). The GF microelectrodes showed a CSC_c_ ~ 2–3 orders of magnitude higher than that of the PtIr electrodes with the same diameter (889.8 ± 158.0 mC cm^−2^ vs. 2.1 ± 0.7 mC cm^−2^, mean ± SD, *n* = 5). Voltage transient measurements were carried out to estimate the charge-injection-limit (CIL), which is defined as the maximum charge that can be injected in a current-controlled stimulation pulse without polarizing an electrode beyond the potentials for water reduction or oxidation^22^. The GF microelectrodes have a wide water window of −1.5 to 1.3 V vs. Ag/AgCl (Supplementary Fig. 2). We estimated the CIL of the GF electrodes using −1.5 V as the threshold (see “Methods” section and Supplementary Fig. 3 for details). The GF microelectrodes exhibited a CIL of 10.1 ± 2.25 mC cm^−2^ (mean ± SD, *n* = 5), which is higher than most commonly used electrode materials for neural stimulation, including PtIr, titanium nitride, iridium oxide, and carbon nanotube (CNT) fiber (Fig. 1h)^22–26^. The conducting polymer poly(3,4-ethylenedioxythiophene) PEDOT is reported to possess the highest charge-injection-capacity due to both Faradaic and non-Faradaic mechanisms at the PEDOT–electrolyte interface, and is extensively utilized to coat electrodes to improve their electrochemical performance. The CIL of the GF microelectrodes is slightly lower than that of PEDOT. However, the PEDOT coating on electrodes is reported to suffer from chemical degradation, delamination, and cracks, which makes it incapable of stable chronic stimulation^27,28^. In our test, we also observed a dramatic impedance increase of the PEDOT-coated PtIr electrodes after 16 days of continuous pulsing at an overcurrent condition (a total of 172.8 M current-controlled pulses at 1 mA), indicating degradation of the PEDOT coating (Supplementary Fig. 4). Conversely, the GF electrodes displayed stable impedance and CIL values, even when subjected to more cycles of overcurrent pulsing (a total of 205.2 M pulses, 19 days) (Fig. 1i). Moreover, continuous monitoring of impedance of the GF electrodes in vivo revealed nearly constant values over time for up to 24 days (Supplementary Fig. 5). These results indicate the high stability of the GF electrodes.

In clinical settings, DBS targeting the selected brain region is now extensively employed for the treatment of various intractable neurological and neuropsychiatric disorders^5^. Efficacious and safe electrical stimulation not only requires high charge-injection-capability but also stability from electrode materials. The GF electrodes show a higher CIL than most available electrode materials, and higher stability than PEDOT-modified metal electrodes. We attribute the high charge-injection-capacity of the GF electrodes to the porous structure and large surface area of their active stimulation sites that are accessible to ions. The high charge-injection-capacity of the GF electrodes allows for the use of small electrodes without the loss of stimulation efficacy, which is not only important to maintain a small MRI artifact size but could also activate a comparatively smaller population of neurons, thus improving the spatial resolution and selectivity of the neural stimulation^20^.

### STN–DBS in hemi-Parkinsonian rats using GF microelectrodes

**Fig. 2:**
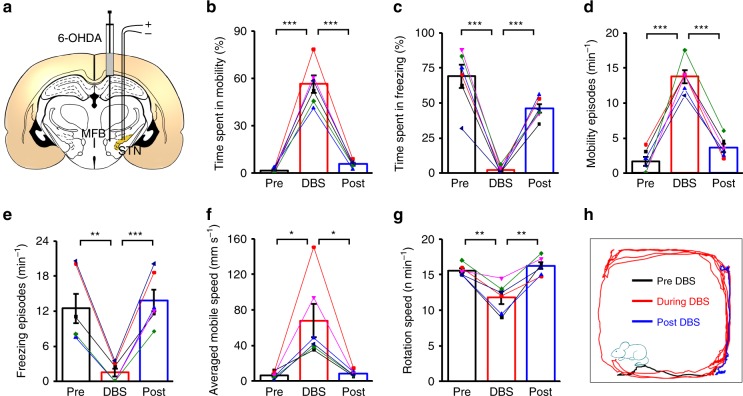
STN–DBS with GF bipolar electrodes alleviates Parkinsonian motor deficits in 6-OHDA-lesioned rats. **a** A schematic section showing the placement of the GF bipolar stimulating electrodes at the STN ipsilateral to the 6-OHDA lesion. **b**–**e** Quantification of the locomotor activities of the hemi-Parkinsonian rats, including the time spent in mobility (**b**), time spent in freezing (**c**), mobile episodes per minute (**d**), and freezing episodes per minute (**e**) before (Pre), during (DBS), and after (Post) STN–DBS with GF bipolar electrodes. **f** Analysis of the average mobile speed of the hemi-Parkinsonian rats before (Pre), during (DBS), and after (Post) STN–DBS with GF bipolar electrodes. **g** Analysis of the apomorphine-induced contralateral rotation speed (in number of turns per min) before (Pre), during (DBS), and after (Post) STN–DBS with GF bipolar electrodes. **h** An example of the locomotor activity of a hemi-Parkinsonian rat before (black line, 2 min), during (red line, 2 min), and after (blue line, 2 min) STN–DBS with a GF bipolar electrode. Data from the same animals are connected with lines and distinguished by color in **b**–**g**. Data represented as mean ± SEM (*n* = 6 animals, *p < 0.05; **p < 0.01, ***p < 0.001, two-tailed paired *t* test). Source data are provided as a Source Data file.

We demonstrated the capability of the GF microelectrodes for efficacious DBS in a hemi-Parkinsonian rat model. The hemi-Parkinsonian model was generated by unilateral injection of the neurotoxin 6-hydroxydopamine (6-OHDA) into the medial forebrain bundle (MFB) of the adult rat brains, resulting in a loss of dopamine neurons, which models Parkinson’s disease on the contralateral side of the body^29^. Successful generation of the hemi-Parkinsonian rat model was confirmed by the apomorphine-induced contralateral rotation test^30^. Bipolar GF microelectrodes were implanted in the ipsilateral STN, a common DBS target for the treatment of Parkinson’s disease^5,29^ (Fig. 2a). For each subject, electrode tip placements within the STN were verified by T_2_-weighted rapid acquisition with relaxation enhancement (RARE) anatomical MRI images acquired immediately after the implantation, and haematoxylin eosin (H&E) staining of the coronal brain sections at the end of the study (Supplementary Fig. 6). Those with electrode tips outside of STN were discarded from the study. The artifact-free property of the GF electrodes, as is described in the following section, could enable more precise in vivo localization and placement verification of the implanted electrodes.

High-frequency stimulation consisting of 130 Hz square constant current pulses (biphasic, symmetric, charge-balanced pulses at 50–200 μA and 60 μs width per phase) was applied to the GF bipolar microelectrodes implanted in rats with PD. Open-field tests showed that these rats (*n* = 6) spent 1.8 ± 0.6% of their time mobile, and 69.0 ± 8.3% of their time freezing. When STN–DBS was turned on, these rats spent more time mobile as 56.4 ± 5.4% and less time freezing as 2.3 ± 1.0% (both *p* < 0.001 compared with DBS off) (Fig. 2b, c). The improved mobility proved the therapeutic efficacy of STN–DBS with GF electrodes. When STN–DBS was turned off, the time spent mobile and freezing immediately changed back to 5.6 ± 0.9% (*p* < 0.001 compared with DBS on) and 46.2 ± 3.2% (*p* < 0.001 compared with DBS on), respectively, indicating the disappearance of the above beneficial effects. The improved mobility of these rats in the open arena by STN–DBS with GF electrodes was also confirmed by the change in the episode number spent in mobility and freezing (Fig. 2d, e). Bradykinesia symptoms were also alleviated by STN–DBS with GF electrodes, as indicated by the increase of mobile speed from 6.3 ± 1.6 mm s^−1^ to 67.9 ± 18.7 mm s^−1^ (*p* < 0.05 compared with DBS off). Mobile speed changed back to 8.5 ± 1.3 mm s^−1^ (*p* < 0.05 compared with DBS on) when STN–DBS was turned off (Fig. 2f). Furthermore, in the apomorphine-induced contralateral rotation test, a statistically significant reduction of rotation speed was observed during STN–DBS with the GF electrodes (Fig. 2g). An example of the locomotor activity of a hemi-Parkinsonian rat before, during, and after STN–DBS with GF electrodes is shown in Fig. 2h. These results confirmed the therapeutic efficacy of the DBS with GF microelectrodes in activating the STN pathway and alleviating motor deficits of the hemi-Parkinsonian animals.

### In vivo assessment of MRI compatibility

**Fig. 3:**
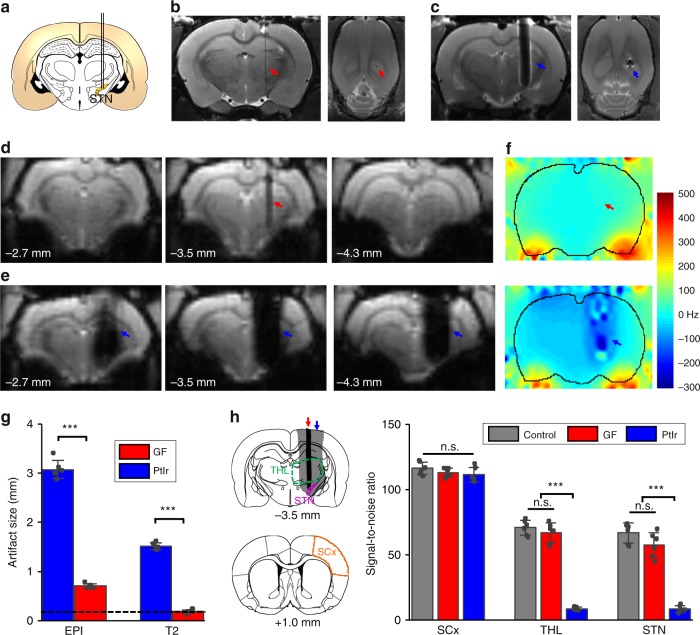
In vivo assessment of MRI artifact. **a** A schematic section showing the placement of the electrodes at the STN of rat brains in MRI artifact studies. Each bipolar electrode was composed of a pair of GFs or PtIr wires (75 μm diameter) insulated with ~5-μm-thick Parylene. Thus, the actual size in the medial–lateral direction was ~170 μm. **b**, **c** Representative coronal (left) and horizontal (right) sections of the T_2_ MRI images of rat brains implanted with a GF (**b**) and PtIr (**c**) bipolar microelectrode, through the position of the implants. **d**, **e** Representative three serial coronal scans from rostral (left) to caudal (right) of EPI images from rat brains implanted with a GF (**d**) and PtIr (**e**) bipolar microelectrode, with the middle images depicting the electrode implant sites. The numbers in each image denote the relative distance from bregma. **f** B0 distortion maps observed in rats implanted with a GF (upper) and PtIr (lower) bipolar electrode. Red and blue arrows in **b**–**f** point to the GF and PtIr implants, respectively. **g** MRI artifact size of the GF and PtIr bipolar electrodes. The black dashed line denotes the actual size of the bipolar electrodes. Data represented as mean ± SD (*n* = 6 electrodes, ***p < 0.001, two-tailed unpaired *t* test). **h** SNR of the EPI signal in several brain areas in control rats without any implant, and rats implanted with GF and PtIr bipolar electrodes at the STN. The ROIs of the three tested brain nuclei were defined from single slices, as indicated in the left reference diagrams. The top diagram represents the implantation plane, and the red and blue arrows point to the EPI artifact outline of the GF and PtIr bipolar electrodes, respectively. The numbers below the diagrams denote their relative distance from bregma. Somatosensory cortex (SCx) = orange; thalamus (THL) = green; STN = purple. Data represented as mean ± SD (*n* = 6 samples, n.s.: not significant; ***p < 0.001, one-way ANOVA tests with Tukey post hoc analysis). Source data are provided as a Source Data file.

We evaluated the MRI artifacts of the GF microelectrodes in a high-field 9.4 T MRI scanner and compared them to those made of PtIr, which is the material most commonly used in clinical neural stimulation devices^31,32^. The same GF and PtIr microwire diameter (75 μm) was used. The electrodes were implanted in the STN of the rat brains (Fig. 3a). A canny edge detector was used to detect the artifact edge, from which the artifact size was measured (Supplementary Fig. 7). The GF bipolar electrodes showed an artifact with a size of 0.18 ± 0.04 mm (*n* = 6), comparable with their actual size of ~0.17 mm and much smaller than that from the PtIr bipolar electrodes (1.51 ± 0.07 mm, *n* = 6) in the T_2_ anatomical images (Fig. 3b, c, g). Because the echo-planar imaging (EPI) sequence, commonly used in fMRI, is more sensitive to the susceptibility mismatch, both electrodes exhibited larger artifacts in the EPI images (Fig. 3d, e) than the anatomical T_2_ images, which makes the fMRI particularly vulnerable to influences from field distortion caused by the implants. The GF bipolar electrodes displayed an artifact of 0.70 ± 0.05 mm (*n* = 6) in the EPI images, while the PtIr bipolar electrodes showed a much more pronounced artifact with sizes of 3.08 ± 0.18 mm (*n* = 6). Such a large volume of signal dropout clearly obstructed a significant portion of the total rat brain area, resulting in a loss of functional response visualization during MRI scans (Fig. 3e). The large artifact of the PtIr electrodes was also manifested in serial slices from rostral (left) to caudal (right) of the EPI images, in which a total of six slices showed the artifact; whereas, for the GF electrodes, their artifact was visible in one slice and was slightly shown in one adjacent slice (Fig. 3d, e; Supplementary Fig. 8). In addition, we performed MRI scans on rat brains implanted with the GF bipolar electrodes under the application of electrical stimulation pulses, and no difference was observed in electrode artifact size for either T_2_-weighted or EPI images (Supplementary Fig. 9), indicating that the application of the electrical pulses will not induce additional artifacts.

We outlined and overlaid the EPI artifacts of both electrodes on reference diagrams showing the coronal section of the rat atlas (Supplementary Fig. 8). It can be seen that a significant number of brain nuclei were overlapped with the blind regions surrounding the PtIr bipolar electrodes (a full list of these nuclei can be found in Supplementary Table 1), with some of them almost entirely blocked by the artifact. These affected brain regions will either be inaccessible or give a low response amplitude in fMRI mapping, leading to an incomplete and biased activation pattern map. Distinctly, for GF bipolar electrodes, no additional brain nuclei—except for those overlapped with the actual electrode tracks—were affected. All of the regions affected by the GF bipolar electrode artifact still had significant portions outside of the artifact, which made them all accessible to fMRI. EPI signal-to-noise ratios (SNRs) of some examined basal ganglia–thalamocortical regions, including the somatosensory cortex, thalamus, and even the target STN in brains implanted with GF bipolar electrodes, showed no significant difference compared with those in control animals with no implants. In contrast, EPI SNRs from brains implanted with PtIr bipolar electrodes were significantly attenuated for the thalamus and STN (Fig. 3h). No significant influence was found in the somatosensory cortex due to its large distance from the implants. Furthermore, B0 maps of rat brains implanted with the GF bipolar electrodes did not reveal any detectable field distortions around the implant, differing from the PtIr electrode implants which caused obvious and extensive B0 field variation around the implants (Fig. 3f). These quantitative results demonstrated that the GF electrodes caused minimal interference to the magnetic field, and their presence would not cause significant attenuation in fMRI signals, thus enabling a full and unbiased mapping of the activation pattern under DBS–fMRI studies. Such advantage is critical for exploring the neuromodulatory effects and mechanisms of DBS therapies. It was noted that the GF electrodes on one-segment EPI images showed comparable artifact size as that on four-segment EPI images (Supplementary Fig. 10), therefore ensuring high MRI compatibility and full activation pattern mapping capability for most fMRI studies.

Tungsten wire and carbon fiber (CF) electrodes were also used previously in simultaneous DBS–fMRI studies^9,13^. Bipolar electrodes made from 75-µm diameter tungsten wires showed EPI artifact of 2.14 ± 0.24 mm under 9.4T MRI (Supplementary Fig. 11), which is slightly smaller than that from PtIr electrodes of the same size (3.08 ± 0.18 mm), and still significantly larger than that from GF electrodes of the same size (0.70 ± 0.05 mm). CF electrodes of a similar size exhibited an EPI artifact size of 0.85 ± 0.14 mm, which was comparable with that from GF electrodes (Supplementary Fig. 11). However, we found that CF electrodes had a very low charge-injection-capacity (CIL of 0.05 mC cm^−2^ vs. 10.1 mC cm^−2^ for GF electrodes), which is consistent with previous reports^20^. Due to this very low charge-injection-capacity, a much larger electrode size is required to inject sufficient charge for DBS to elicit the desired physiological response without damages to tissues or electrodes (Supplementary Fig. 12), which would in turn increase artifact sizes for DBS–fMRI. Supplementary Table 2 lists the measured CIL, and calculated minimum wire diameters *d*_min_ for electrodes required to inject 200-µA current pulses without polarizing electrode potential beyond the water window, for various materials, including GF, CF, PtIr, tungsten, and graphene-encapsulated copper (G–Cu)^33^. EPI images from rat brains implanted with various bipolar electrodes made of wires with this minimum wire diameter *d*_min_ are shown in Supplementary Fig. 13. Under the prerequisite of safe charge injection without electrode potential excursion beyond the water window, the CF electrodes led to a ~2× EPI artifact size compared with the GF electrodes used in our study (with diameter of 75 µm, Supplementary Fig. 14), which caused significantly reduced SNR for some nuclei close to the electrodes, including thalamus and STN (Supplementary Fig. 14). Among all tested materials, GF was the only one that did not cause signal loss for either thalamus or STN (Supplementary Fig. 14), thus possessing the capability for full activation pattern mapping. In addition, the large size of the CF bipolar electrodes (~586 μm, Supplementary Figs. 12, 13 and Supplementary Table 2) would also decrease stimulation selectivity, as well as lead to severe acute tissue damages and sustained chronic inflammatory responses^20,34^, all of which are undesirable for neural electrical modulation.

### DBS–fMRI studies

**Fig. 4:**
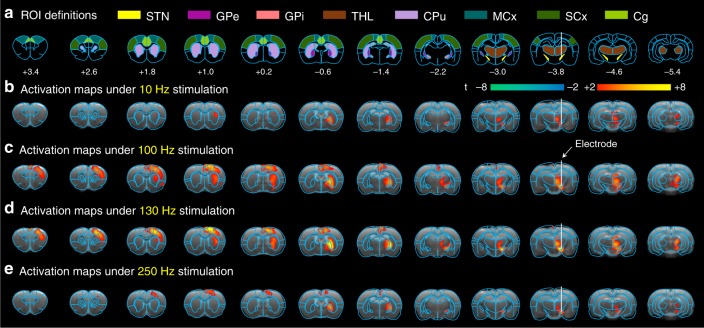
BOLD activation maps evoked by STN–DBS with GF electrodes in PD rats. Four stimulation frequencies were tested as marked in each panel. The BOLD activation maps are overlaid onto averaged anatomical images. Numbers below slices denote relative distance from bregma (in mm). The same set of distance numbers applies to the slices in **b**–**e**. Color bar denotes t-score values obtained by GLM analyses, with a significance threshold of uncorrected *p* < 0.001. All data are group averaged, *n* = 24 scans from eight rats. STN subthalamic nucleus, GPe external globus pallidus, GPi internal globus pallidus, THL thalamus, CPu caudate putamen, MCx motor cortex, SCx somatosensory cortex, Cg cingulate cortex.

**Fig. 5:**
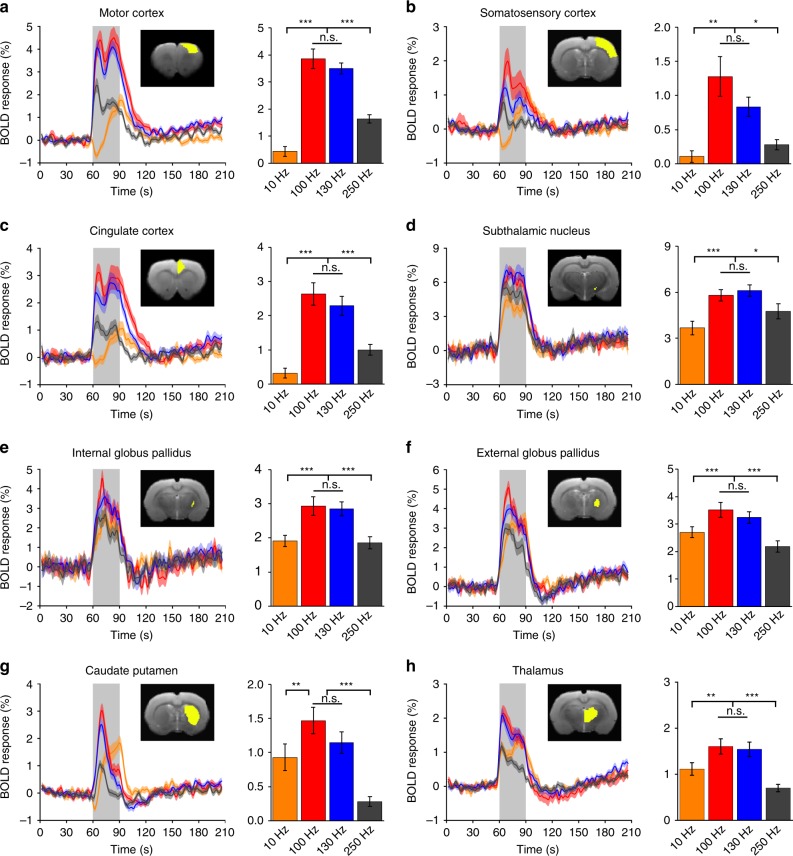
BOLD signal time series at selected anatomically defined ROIs evoked by STN–DBS with GF electrodes in PD rats. Percent BOLD response over time at each ROI is shown for multiple stimulation frequencies (orange, 10 Hz; red, 100 Hz; blue, 130 Hz; gray, 250 Hz). The stimulation epoch is indicated by a gray-shaded band. The solid lines show the average signal, and the shaded regions represent the SEM, *n* = 24 from eight rats. The bar graphs display the average percent changes in BOLD amplitude during the stimulation period. Data represented as mean ± SEM (*n* = 24 scans from eight rats, n.s.: not significant; *p < 0.05; **p < 0.01; ***p < 0.001, one-way repeated measures ANOVA tests with Tukey post hoc analysis). The inserts depict representative slice examples for each predefined ROI (note that most ROIs encompassed multiple slices). All ROIs are ipsilateral to the DBS hemisphere. Source data are provided as a Source Data file.

**Fig. 6:**
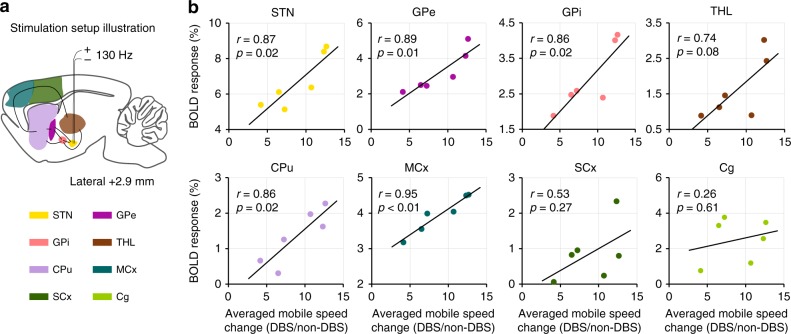
Correlation between BOLD responses and mobile speed change. **a** A schematic section showing the placement of the stimulating electrode at STN. **b** Scatter plot between regional BOLD responses and averaged mobile speed change under 130 Hz STN–DBS. The averaged mobile speed change is defined as averaged mobile speed under DBS divided by that without DBS (non-DBS), which is the average of pre- and post-DBS values. The Pearson’s correlation coefficient *r* between mobile speed increase and BOLD responses across rats was calculated for each ROI. Source data are provided as a Source Data file.

The STN–DBS using GF bipolar electrodes in hemi-Parkinsonian rats evoked significant and frequency-dependent positive BOLD responses in the ipsilateral basal ganglia–thalamocortical network, including multiple cortical and subcortical regions (Fig. 4). Individual BOLD activation maps from each rat are included in Supplementary Fig. 15. No significantly modulated voxels were observed in the contralateral hemisphere. DBS–fMRI measurements of the same rats immediately after sacrifice revealed no BOLD responses (Supplementary Fig. 16). This suggests that the BOLD responses that we observed here reflected brain activities, rather than artifact caused from the electrical stimulation. In addition, the time courses of several anatomical regions of interest (ROIs) were calculated for each stimulation frequency, which demonstrated clear BOLD signal changes that were time-locked to the stimulation pulse blocks (Fig. 5). The maximal amplitude of the BOLD signals was observed at 100 and 130 Hz, which are the therapeutic effective frequencies used in clinical settings. Of all of the regions examined, the DBS target STN showed the largest percentage of BOLD changes (~5.81 ± 0.36% at 100 Hz, and 6.12 ± 0.38% at 130 Hz DBS, *n* = 24). Among the examined cortical regions, the motor cortex exhibited the largest BOLD signal changes (3.86 ± 0.36% at 100 Hz, 3.50 ± 0.21% at 130 Hz DBS, *n* = 24), and the somatosensory cortex showed the lowest responses (1.28 ± 0.29% at 100 Hz, 0.84 ± 0.14% at 130 Hz DBS, *n* = 24). A close examination of the signal time traces revealed a clear “double peak” feature of the BOLD signal in certain regions, including the motor cortex, somatosensory cortex, cingulate cortex, and STN (Fig. 5), possibly due to the involvement of two different circuitries or a delayed neurotransmission effect^10^. It is worth noting that, for 10 Hz STN–DBS, the three cortex and caudate putamen regions first produced a small negative BOLD signal followed by a positive BOLD response during the stimulation epoch, a feature that is distinct from other regions and other frequencies. This suggests the possibility of a biphasic neuronal response^35^. Importantly, the BOLD activation within STN, GPi, GPe, caudate putamen, and motor cortices exhibited significant correlations with the mobile speed increase of the hemi-Parkinsonian rats under 130 Hz DBS (*p* < 0.05, Fig. 6).

## Discussion

Simultaneous DBS and fMRI serves as a powerful tool for examining the modulatory effects of electrical stimulation on brain network activity in vivo, which is important to understand the underlying therapeutic mechanism of DBS. The little-to-no artifact of the GF electrodes makes the fMRI mapping accessible to all brain regions, thus providing a way for full and unbiased activation pattern mapping under DBS, especially in small animal studies. Previous work on DBS–fMRI at the STN target in rats using conventional stimulating electrodes, such as PtIr, failed to demonstrate reliable BOLD responses within regions in the STN, internal and external globus pallidus (GPi and GPe), and thalamic nuclei, due to the electrode artifact^11^. Even for human DBS–fMRI investigations, it was reported that the electrode artifact could lead to regional bias in activation pattern mapping^36,37^.

With the utilization of GF electrodes, we observed that STN–DBS in hemi-Parkinsonian rats modulated the activity of the basal ganglia–thalamocortical network in a frequency-dependent manner. Robust positive BOLD responses to STN–DBS were observed in both distant and local areas, including the motor cortex, somatosensory cortex, cingulate cortex, GPi, GPe, caudate putamen, thalamus, and even the DBS target of STN. This activation pattern indicates the modulation of both motor and non-motor pathways by STN–DBS. The strong positive activation of the ipsilateral motor cortex was consistent with the therapeutic motor effects of DBS. Prior work demonstrated direct antidromic activation of primary motor cortex neurons during STN–DBS using electrophysiological studies^30^. Optogenetic research showed that the therapeutic motor effects were from antidromic stimulation of the afferent hyperdirect pathway from the cortex to STN, rather than from orthodromic projections to GPi^29^. The significant correlations of BOLD activation within STN, GPi, GPe, caudate putamen, and motor cortices with the mobile speed increase of the hemi-Parkinsonian rats observed here indicated that the alleviation of PD symptom under STN–DBS was related to the modulation of the basal ganglia. These results suggest that both orthodromic stimulation of the feed-forward thalamocortical circuit and antidromic activation of motor cortical afferents might underlie the therapeutic mechanism of STN–DBS.

The activation in the somatosensory and cingulate cortex indicate the involvement of non-motor circuits which may be related to the sensory and limbic effects observed clinically for STN–DBS, particularly the effects on pain and mood^38,39^. An early human DBS–fMRI case study reported that, in one patient, depressive dysphoria was induced by right-side DBS accompanied by positive BOLD activation in the superior prefrontal cortex, anterior cingulate, and anterior thalamus, with the implanted electrode marginally superior and lateral to the intended STN target^40^. Another investigation showed that stimulation applied at the anteromedial STN contact and the contact immediately dorsal to it consistently produced a hypomanic state, in addition to an alleviation of motor symptoms. Their positron emission tomography (PET) study revealed the activation of limbic and association cortex, including areas of the anterior cingulate gyrus and ventral anterior nucleus of the thalamus^41^. These side effects are understandable because STN is known to be involved in multiple circuits connecting cortical regions to the basal ganglia that regulate motor, cognitive, and emotional behavior^42^. These studies also showed that the exact position of the stimulation sites within different STN sub-regions might affect both the motor and non-motor outcome of DBS. We believe that, for future human applications, the artifact-free property of the GF electrodes under anatomical MRI and their small size would enable more precise MR-guided electrode implantation to minimize location inaccuracies, thus assisting to obtain optimal clinical benefits and alleviate adverse side effects from DBS.

The activation of caudate putamen, thalamus, and globus pallidus were also observed in previous human and animal DBS–fMRI studies. Greater activation in caudate putamen was reported in normal anesthetized pigs when using STN as a DBS target than using a GPi target, which might help to understand the fact that more patients were able to reduce medication dosage using STN–DBS than using GPi-DBS^43^. In a human pilot study, it was reported that the stimulation was effective over a longer period of time for patients with BOLD signal activation observed in the globus pallidus than those exhibiting no activation in the pallidum^44^. While determination of how the activation of various brain areas correlates with the therapeutic effects of DBS warrants additional research, we believe that DBS–fMRI may act as an effective paradigm in rating the clinical effect of the procedure. One limitation of the present work is that the difference in states between behavioral testing (awake) and fMRI (anesthetized) might limit our ability to detect all potential neural correlates of the DBS therapeutic effect. For future studies, applications of awake rat fMRI^45^ would be beneficial to provide more detailed clues of circuitry mechanisms of DBS therapies.

Our work demonstrates the advantage of GF electrodes in simultaneous DBS and fMRI studies. We believe that the little-to-no artifact of the GF electrodes is attributable to the close magnetic susceptibility between the GFs and water/tissues. It was observed that artifacts from all electrodes were much more evident on the functional images (T_2_*-weighted gradient-echo EPI images) compared with the structural images (T_2_-weighted RARE images) (Fig. 3b–e), which indicates that the artifacts are primarily resultant from magnetic field inhomogeneity as the gradient-echo EPI images are more sensitive to magnetic susceptibility inhomogeneity than the T_2_-weighted RARE images^46^. The PtIr electrodes severely degraded MRI image quality with a relatively large paramagnetic susceptibility of ~231 ppm, and *∆χ* ≈ 240 ppm with respect to that of water (*χ* = −9.05 ppm)^47^. The tungsten electrodes of the same size showed less artifact than PtIr electrodes with magnetic susceptibility of ~77.2 ppm, and *∆χ* ≈ 86 ppm with respect to that of water^14^. Although the precise value of the overall magnetic susceptibility of the GFs is not yet available, their little-to-no artifact suggests a close value to that of water. Eddy currents can be induced in implantable electrodes by gradient switching and RF field, which might contribute to the MRI artifact. However, because the area receiving the magnetic flux is small as a result of small electrode diameter, the induced eddy currents in GFs used here were small and decayed rapidly (see Supplementary Information for details), which makes the eddy current origin negligible for the MRI artifact^26^. Furthermore, the interactions between the MRI environment and conductive implants pose risks of electrode heating and tissue damage^48^. The H&E staining results showed no obvious tissue damage around the electrode tips after the DBS–fMRI studies under the conditions used here. This indicates that, under strict guidelines, the DBS–fMRI can be safe for future human applications.

In conclusion, our DBS–fMRI study using GF electrode technology revealed a full activation pattern under STN–DBS in Parkinsonian rats, which is not achievable by other metal or CF electrodes. The DBS–fMRI studies with the GF electrodes are widely applicable to other targets or neurological conditions. With the unique capability for full and unbiased mapping of the entire circuit and network connectivity without obstructing brain nuclei, future DBS–fMRI studies with the GF electrodes at different targets and with varied stimulation frequency and strength could provide important insights into brain circuitries and network connections, as well as the therapeutic mechanisms underlying various DBS therapies.

## Methods

### Microelectrode fabrication and characterization

Graphene fibers (GFs) were prepared through a one-step dimensionally confined hydrothermal process using suspensions of graphene oxide (GO) (monolayer, thickness: 0.8–1.2 nm; sheet diameter: 0.5–5 µm; #XF002-2, Nanjing/Jiangsu XFNANO Materials Technology, China). In a typical preparation, an 8 mg mL^−1^ aqueous GO suspension was injected into a glass pipeline with a 0.9 mm inner diameter using a syringe. After being baked at 230 °C for 2 h with the two ends of the pipeline sealed, a GF matching the pipe geometry was produced. This preformed GF was then released from the pipeline by flow of N_2_ and dried in air. The dried GF had a reduction in diameter to ~75 μm due to water loss and drying-induced alignment of the GO sheets. Measurements of the Young’s modulus of these GFs using a single-column testing instrument (Instron 5843, USA) gave an average Young’s modulus of 2–3 GPa. The diameter of 75 μm was used in all studies in this work. The GF samples were characterized by scanning electron microscopy (Hitachi S-4800 operated at 1–2 kV acceleration voltage, Japan) and Raman spectroscopy (Jobin-Yvon Horiba LabRAM HR-800, 514 nm, ×100 objective, France, LabSpec Version 5.36.11). Parylene-C film of 5 μm thickness was deposited onto the GFs in a homemade low-pressure coating system. To make a bipolar GF-stimulating electrode, a pair of GFs with Parylene-C insulation were aligned in parallel and mechanically pasted together with glue. One end of two GFs was soldered onto a custom-made MR compatible female header connector made of high-purity copper. A sharp blade was used to cut the GFs to the desired length and expose the cross sections of the pair of GFs as the active stimulating sites. The same method described above was used to fabricate other bipolar electrodes from PtIr microwires (#767600, A-M Systems, USA), tungsten microwires (#797550, A-M Systems, USA), CFs and G–Cu wires. In all, 1 K tow carbon fiber (#CF701, The Composites Store, USA) was split to produce bundles with desired diameters. G–Cu wires were made by chemical vapor deposition of graphene on copper wires^33^.

All electrochemical measurements were performed in 1× phosphate buffered saline (PBS) with pH 7.4 at room temperature. Electrochemical impedance spectroscopy (EIS) and cyclic voltammetry (CV) were done using a CHI660e electrochemical workstation (version 15.08, CH Instruments, USA). A three-electrode configuration was used, with the tested sample as the working electrode, an Ag/AgCl electrode as the reference electrode, and a large surface area platinum as the counter electrode. CV tests were performed by sweeping the potential of the electrode at a scan rate of 50 mV s^−1^. Each sample was swept for two cycles, and the cathodic charge-storage-capacity (CSCc) was calculated as the time integral of the cathodic current recorded over a potential range of −0.6 to 0.8 V in the second cycle. For water window testing, CV was performed between the voltage limits of −1.8 to 1.8 V at a scan rate of 1 mV s^−1^. The water window of the GF electrodes was determined as the water oxidation and reduction potential obtained from CV measurements, where a steep increase in current was observed (Supplementary Fig. 2).

For voltage transient experiments, a three-electrode cell (the same as above) was used. Biphasic, symmetric, and charge-balanced current pulses of 60 μs duration (Supplementary Fig. 3) were delivered to the tested sample at a frequency of 130 Hz with a stimulator (Model 2100, A-M Systems, USA). Voltage transients under the current pulses were recorded with an oscilloscope, and the negative potential excursion (V_exc_) was calculated by subtracting the initial access voltage (V_acc_) due to solution resistance from the total voltage (V_tot_, Supplementary Fig. 3). The charge-injection-limit was calculated by multiplying the current amplitude and pulse duration at which V_exc_ reaches the water reduction limit (−1.5 and −0.6 V for GF electrodes and PtIr electrodes, respectively), divided by the geometric surface area of the electrodes.

Stability testing under continuous overcurrent pulsing was performed by immersing the GF electrodes in a cell filled with 1× PBS, pH 7.4 at room temperature. A two-electrode configuration was used with the GF electrode as the working electrode, and a large surface area Pt foil electrode used as the return and reference electrode. The cell was sealed in order to avoid evaporation of the electrolyte, and thus keep the solution impedance constant. The electrodes were tested prior to the beginning of the stability experiments (day 0), and on each of the following days after ~23 h of continuous stimulation (10.8 M pulses day^−1^, 130 Hz, biphasic, symmetric, and charge-balanced current pulses of 60 μs duration) at 1 mA amplitude. This pulse amplitude is larger than what is commonly used in DBS for PD (50–300 μA). The experiment was concluded after 19 days of continuous stimulation.

The same protocol described above was used to test the stability of the PEDOT-poly (styrene sulfonate) (PSS) deposited on the electrically active sites of the PtIr microelectrodes. For PEDOT-PSS deposition, electrolyte consisting of 0.01 M 3,4-ethylenedioxylthiophene (EDOT) (Sigma-Aldrich, USA) and 0.1 M sodium PSS (Sigma-Aldrich, USA) aqueous solution was used. The electrochemically polymerized reaction was performed in a three-electrode cell under galvanostatic conditions. A platinum foil was used as the counter electrode, and an Ag/AgCl electrode was used as the reference electrode. In the galvanostatic mode, the polymerization was carried out under a constant current of 22 nA for 30 min. After PEDOT-PSS deposition, samples were kept immersed in deionized water for 2 h to remove impurities and excess EDOT. The electrodes were tested before the PEDOT-PSS deposition and on the same day of the PEDOT-PSS deposition (day 0).

### Animal surgery

Adult male Sprague-Dawley rats weighing 250–280 g (Charles River Laboratories, China) were used throughout this study. Our procedures for handling the animals complied with the Beijing Administration Rules of Laboratory Animals and the National Standards of Laboratory Animal Requirements of Environment and Housing Facilities (GB 14925−2010), and were approved by the Institutional Animal Care and Use Committee of Peking University. For surgery, the rats were anesthetized using constant 2–2.5% isoflurane in medical-grade oxygen. Rats were secured in a stereotactic apparatus (Lab Standard Stereotaxic Instrument, Stoelting, USA) throughout the procedure. The hemi-Parkinsonian rats were generated by unilateral injection of 6-hydroxydopamine (6-OHDA, 1.6 μL, 5 mg mL^−1^ dissolved in 0.9% saline; Sigma-Aldrich, USA) into the medial forebrain bundle (MFB) (AP: −4.4 mm; ML: −1.1 mm; DV: 8.0 mm from dura). After a 2-w recovery period, apomorphine-induced contralateral rotation behavior was tested to confirm whether the hemi-Parkinsonian rat model was successfully induced. Each rat was injected with apomorphine (0.5 mg kg^−1^, Sigma-Aldrich, USA) subcutaneously. After 15 min, the contralateral rotational number was counted for 5 min, and those exhibiting a contralateral rotation speed exceeding 15 turns min^−1^ were considered as successful based on previous similar studies and selected for electrode implantation.

In a typical implantation of the DBS electrodes, a bipolar GF microelectrode was implanted unilaterally into the STN (AP: −3.5 mm, ML: −2.5 mm, DV: 7.8 mm from dura) of a hemi-Parkinsonian rat. All GF microelectrodes used in this study were directly inserted into the rat brains. Craniotomies were sealed with a silicone elastomer (World Precision Instruments, USA). Ceramic bone anchor screws, together with dental methacrylate, were used to fix the connector and electrode set onto the rat skull. Electrode tip placements within the STN were verified for each subject by T_2_-weighted RARE anatomical MRI images acquired immediately after implantation and H&E staining of the coronal brain sections at the end of the study. Animals with electrode placements outside of the target regions were discarded from the study and excluded from all further experimental analyses. They were also not included in the final subject numbers.

### DBS and behavioral tests

The efficacy of the GF microelectrodes for DBS was assessed by open-field testing on hemi-Parkinsonian rats. In a typical test, a rat was placed in a box (75 × 75 cm in square and 40 cm high), and the position of the rat’s body center was tracked using ANY-maze software (Stoelting, USA), with a digital video camera mounted directly above the arena. An electrical commutator and pulley system was used to allow the rat to move and turn freely within the box. A stimulator (Model 2100, A-M Systems, USA) was used to deliver continuous electrical pulses (biphasic, symmetric, and charge-balanced current pulses of 60 μs duration at 130 Hz). The optimal current intensity was determined by the maximum value that did not cause dyskinetic movement of the contralateral forelimb which gave a current range of 50–200 μA, although for most animals, 100 μA was used. This stimulation parameter setting was consistent with those used in clinical settings^49^. The motor performance of the hemi-Parkinsonian rats before (2 min), during (2 min), and after STN–DBS (2 min) was compared. Several motor behavioral indexes, including time spent in mobility, time spent in freezing, mobile episodes, freezing episodes, and average mobile speed, were recorded and analyzed with ANY-maze software (version 4.70)^30^. Rotation speed was counted and calculated manually. The time spent in mobility and freezing did not add up to 100%, because the rats spent the rest of the time in fine movements.

### MRI acquisitions

All MRI experiments were performed in a Bruker 9.4T scanner with Bruker’s 86 mm volume coil for transmission and a 2-cm diameter single-loop surface coil for receiving (ParaVision Version 6.0.1 for MRI acquisitions). The implanted electrodes were bent 90° and laid flat along the rats’ skulls to allow the placement of the MRI surface receiver coil over the rat heads. Rats were anesthetized with 4% isoflurane, followed by a bolus injection of dexmedetomidine (0.022 mg kg^−1^). During MRI scanning, isoflurane (0.5%) delivered via a nose cone combined with continuous infusion of dexmedetomidine (0.015 mg kg^−1^ h^−1^) was used to maintain anesthesia^50^. Animal temperature, respiration, and blood oxygen saturation were all monitored and within normal ranges (Model 1025, SA Instruments, USA). Body temperature was maintained at 37 ± 0.5 °C using a circulated hot water bed and a hot air blower.

T_2_-weighted anatomical images were acquired using RARE sequence with the following parameters: TR/TE = 2500/33 ms, RARE factor = 8, FOV = 30 × 30 mm^2^, matrix = 256 × 256, slice thickness = 0.8 mm, and contiguous 20 slices without gap in the axial direction. All fMRI data were acquired using a 4-shot gradient-echo EPI sequence with the following parameters: TR/TE = 500/13 ms, FOV = 30 × 30 mm^2^, matrix = 80 × 80, flip angle = 55°, repetitions = 105, slice thickness = 0.8 mm, and contiguous 14 slices without gap in the axial direction. B0 distortions were assessed by a high-resolution field map acquired using a dual-echo 3D gradient-echo sequence with the following parameters: TR = 20 ms, TE1 = 1.6 ms, TE2 = 5.2 ms, FOV = 40 × 40 × 40 mm^3^, and matrix = 64 × 64 × 64. To measure the electrode artifact size, raw MRI images with the largest electrode artifact were selected and upsampled from 0.12 × 0.12 × 0.8 mm^3^ to 0.06 × 0.06 × 0.8 mm^3^ voxel resolution. A canny edge detector in Matlab (R2018a, Mathworks, USA) was used to detect the artifact edge (Supplementary Fig. 7). The artifact size in the medial–lateral direction was then measured and averaged over different animal subjects.

The fMRI scans were acquired for 210 s (70 repetitions), during which stimulation was applied in a 60 s-OFF/30 s-ON/120 s-OFF cycle, with the following parameters: bipolar square-wave current with an amplitude of 300 μA, frequency of 10, 100, 130, and 250 Hz, and pulse width of 7.8/f ms, where f is frequency in Hz. Pulse width was varied in this way in order to make the total duration of current delivery over the stimulation period constant. Stimulation frequencies were varied in a pseudo-randomized order, and at each DBS frequency the EPI scan was repeated three times per rat for within subject/session averaging. The electrode location at STN was confirmed with H&E staining of the coronal brain sections at the end of the study. DBS–fMRI studies were carried out on eight rats. Two rats were unable to undergo behavioral tests because their connectors were accidentally damaged before the behavioral tests. All animal experiments were not blinded.

### fMRI data analysis

Data analysis was performed using a custom-written program developed using Matlab (R2018a, MathWorks, USA) and SPM12 (http://www.fil.ion.ucl.ac.uk/). EPI images were first grouped by subject and DBS frequency, and realigned to the first volume of the first session, using a least squares approach and a six-parameter rigid body spatial transformation, and then co-registered to the subject’s own T_2_ anatomical images, which were normalized to a rat brain template. EPI images were spatially smoothed with a full-width half maximum (FWHM) of 0.8 × 0.8 mm. B0 field maps were also co-registered to an anatomical template using their reconstructed magnitude images. After preprocessing, statistical analysis was conducted across subjects using a general linear model with reference to the stimulation paradigm, and the default hemodynamic response function of SPM was used. For each scan, the time series was converted to relative BOLD response (ΔS(*t*)/S0), where ΔS(*t*) was generated by subtracting the mean of pre-stimulation period (S0) of that scan. For four different DBS frequency groups, the *t*-statistic maps of each subject were averaged, respectively, with a significance level set at *p* < 0.001.

For ROIs time course analysis, eight ROIs were anatomically defined and applied to co-registered data, including the cingulate cortex, motor cortex, somatosensory cortex, caudate putamen, internal globus pallidus, external globus pallidus, subthalamic nucleus, and thalamus. The BOLD signal time courses were calculated for each ROI. One-way repeated measures ANOVA tests with Tukey post hoc analysis were conducted to evaluate frequency-dependent responses^10^. Significance level was set at *p < 0.05; **p < 0.01; ***p < 0.001. Data plotting and analysis were performed using Origin 2020.

### Reporting summary

Further information on research design is available in the Nature Research Reporting Summary linked to this article.

## Supplementary information

Supplementary Information

Reporting Summary

## Data Availability

The authors declare that the data supporting the findings of this study are available within the article and its Supplementary Information files or available from the corresponding authors upon reasonable request, including all raw MRI image files. The source data underlying Figs. 1e–i, 2b–g, 3g-h, 5a–h, and 6b, and Supplementary Figs. 1, 2, 3, 4, 5, 10c, 12, and 14 are provided as a Source Data file.

## Source Data

Source Data

## Code availability

Custom code used in this study has been deposited on GitHub.

## References

[1] Kemler MA (2000). Spinal cord stimulation in patients with chronic reflex sympathetic dystrophy. N. Engl. J. Med..

[2] Ryugo DK, Kretzmer EA, Niparko JK (2005). Restoration of auditory nerve synapses in cats by cochlear implants. Science.

[3] Jackson A, Zimmermann JB (2012). Neural interfaces for the brain and spinal cord—restoring motor function. Nat. Rev. Neurol..

[4] Milby AH, Halpern CH, Baltuch GH (2008). Vagus nerve stimulation for epilepsy and depression. Neurotherapeutics.

[5] Benabid AL, Chabardes S, Mitrofanis J, Pollak P (2009). Deep brain stimulation of the subthalamic nucleus for the treatment of Parkinson’s disease. Lancet Neurol..

[6] Ressler KJ, Mayberg HS (2007). Targeting abnormal neural circuits in mood and anxiety disorders: from the laboratory to the clinic. Nat. Neurosci..

[7] Pienaar IS (2015). Deep-brain stimulation associates with improved microvascular integrity in the subthalamic nucleus in Parkinson’s disease. Neurobiol. Dis..

[8] Yang PF (2013). Comparison of fMRI BOLD response patterns by electrical stimulation of the ventroposterior complex and medial thalamus of the rat. PLoS ONE.

[9] Chao THH, Chen JH, Yen CT (2014). Repeated BOLD-fMRI imaging of deep brain stimulation responses in rats. PLoS ONE.

[10] Van Den Berge N (2017). Functional circuit mapping of striatal output nuclei using simultaneous deep brain stimulation and fMRI. Neuroimage.

[11] Lai HY, Younce JR, Albaugh DL, Kao YCJ, Shih YYI (2014). Functional MRI reveals frequency-dependent responses during deep brain stimulation at the subthalamic nucleus or internal globus pallidus. Neuroimage.

[12] Arantes PR (2006). Performing functional magnetic resonance imaging in patients with Parkinson’s disease treated with deep brain stimulation. Mov. Disord..

[13] Shyu BC, Lin CY, Sun JJ, Chen SL, Chang C (2004). BOLD response to direct thalamic stimulation reveals a functional connection between the medial thalamus and the anterior cingulate cortex in the rat. Magn. Reson. Med..

[14] Schenck JF (1996). The role of magnetic susceptibility in magnetic resonance imaging: MRI magnetic compatibility of the first and second kinds. Med. Phys..

[15] Kuzum D (2014). Transparent and flexible low noise graphene electrodes for simultaneous electrophysiology and neuroimaging. Nat. Commun..

[16] Park DW (2014). Graphene-based carbon-layered electrode array technology for neural imaging and optogenetic applications. Nat. Commnun..

[17] Yin R (2018). Soft transparent graphene contact lens electrodes for conformal full-cornea recording of electroretinogram. Nat. Commnun..

[18] Zelin D (2012). Facile fabrication of light, flexible and multifunctional graphene fibers. Adv. Mater..

[19] Xin G (2015). Highly thermally conductive and mechanically strong graphene fibers. Science.

[20] Jancrazio PJ (2017). Thinking small: progress on microscale neurostimulation. Technol. Neuromodulation.

[21] Moon IK, Lee J, Ruoff RS, Lee H (2010). Reduced graphene oxide by chemical graphitization. Nat. Commnun..

[22] Cogan SF (2008). Neural stimulation and recording electrodes. Annu. Rev. Biomed. Eng..

[23] Weiland JD, Anderson DJ, Humayun MS (2002). In vitro electrical properties for iridium oxide versus titanium nitride stimulating electrodes. IEEE Trans. Biomed. Eng..

[24] Cogan SF, Troyk PR, Ehrlich J, Plante TD, Detlefsen DE (2006). Potential-biased, asymmetric waveforms for charge-injection with activated iridium oxide (AIROF) neural stimulation electrodes. IEEE Trans. Biomed. Eng..

[25] Vitale F, Summerson SR, Aazhang B, Kemere C, Pasquali M (2015). Neural stimulation and recording with bidirectional, soft carbon nanotube fiber microelectrodes. ACS Nano.

[26] Lu L (2019). Soft and MRI compatible neural electrodes from carbon nanotube fibers. Nano Lett..

[27] Green RA, Lovell NH, Wallace GG, Poole-Warren LA (2008). Conducting polymers for neural interfaces: challenges in developing an effective long-term implant. Biomaterials.

[28] Cui XT, Zhou DD (2007). Poly (3,4-ethylenedioxythiophene) for chronic neural stimulation. IEEE Trans. Neural Syst. Rehabil. Eng..

[29] Gradinaru V, Mogri M, Thompson KR, Henderson JM, Deisseroth K (2009). Optical deconstruction of parkinsonian neural circuitry. Science.

[30] Li Q (2012). Therapeutic deep brain stimulation in parkinsonian rats directly influences motor cortex. Neuron.

[31] Coffey RJ (2009). Deep brain stimulation devices: a brief technical history and review. Artif. Organs.

[32] Tagliati M (2009). Safety of MRI in patients with implanted deep brain stimulation devices. Neuroimage.

[33] Zhao S (2016). Graphene encapsulated copper microwires as highly MRI compatible neural electrodes. Nano Lett..

[34] Stice P, Gilletti A, Panitch A, Muthuswamy J (2007). Thin microelectrodes reduce GFAP expression in the implant site in rodent somatosensory cortex. J. Neural Eng..

[35] Paek SB (2015). Frequency-dependent functional neuromodulatory effects on the motor network by ventral lateral thalamic deep brain stimulation in swine. Neuroimage.

[36] Tarsy, D., Vitek, J. L., Starr, P. & Okun, M. *Deep Brain Stimulation in Neurological and Psychiatric Disorders* (Humana Press, Totowa, 2008).

[37] Kahan J (2012). Therapeutic subthalamic nucleus deep brain stimulation reverses cortico-thalamic coupling during voluntary movements in Parkinson’s disease. PL0S ONE.

[38] Kim HJ (2009). Chronic subthalamic deep brain stimulation improves pain in Parkinson disease. J. Neurol..

[39] Okun MS (2009). Cognition and mood in Parkinson’s disease in subthalamic nucleus versus globus pallidus interna deep brain stimulation: The COMPARE Trial. Ann. Neurol..

[40] Stefurak T (2003). Deep brain stimulation for Parkinson’s disease dissociates mood and motor circuits: a functional MRI case study. Mov. Disord..

[41] Mallet L (2007). Stimulation of subterritories of the subthalamic nucleus reveals its role in the integration of the emotional and motor aspects of behavior. Proc. Natl Acad. Sci. USA.

[42] Alexander GE, Delong MR, Strick PL (1986). Parallel organization of functionally segregated circuits linking basal ganglia and cortex. Annu. Rev. Neurosci..

[43] Min HK (2012). Deep brain stimulation induces BOLD activation in motor and non-motor networks: an fMRI comparison study of STN and EN/GPi DBS in large animals. Neuroimage.

[44] Jech R (2001). Functional magnetic resonance imaging during deep brain stimulation: a pilot study in four patients with Parkinson’s disease. Mov. Disord..

[45] Han Z (2019). Awake and behaving mouse fMRI during Go/No-Go task. Neuroimage.

[46] Georgi JC, Stippich C, Tronnier VM, Heiland S (2004). Active deep brain stimulation during MRI: a feasibility study. Magn. Reson. Med..

[47] Jiang CQ, Hao HW, Li LM (2013). Artifact properties of carbon nanotube yarn electrode in magnetic resonance imaging. J. Neural Eng..

[48] Johannes BE (2018). Should patients with brain implants undergo MRI?. J. Neural Eng..

[49] Nilsson MH, Jarnlo GB, Rehncrona S (2008). Functional balance performance in patients with Parkinson’s disease after long-term treatment with subthalamic nucleus high-frequency stimulation. Parkinsonism Relat. Disord..

[50] Brynildsen JK (2017). Physiological characterization of a robust survival rodent fMRI method. Magn. Reson. Imaging.

