# Supplementary material for: Full activation pattern mapping by simultaneous deep brain stimulation and fMRI with graphene fiber electrodes
Source: Nat Commun. 2020 Apr 14;11:1788. doi: 10.1038/s41467-020-15570-9 (PMC7156737; doi:10.1038/s41467-020-15570-9)
Supplement: Supplementary file 1 — Supplementary Information [file 41467_2020_15570_MOESM1_ESM.pdf]

Supplementary Information for

Full activation pattern mapping by simultaneous deep brain stimulation and fMRI with graphene fiber electrodes

Zhao et al.

Supplementary Methods

Eddy current calculation[1]. Eddy current is resulted from time-varying magnetic field B[1]. We calculated the maximum eddy current I_{max} for both GF fiber and PtIr electrodes with radius R and length h under a 9.4 T MR radio frequency coil. In our case, length h is approximately 0.8 cm; radius R is 37.5 μm.

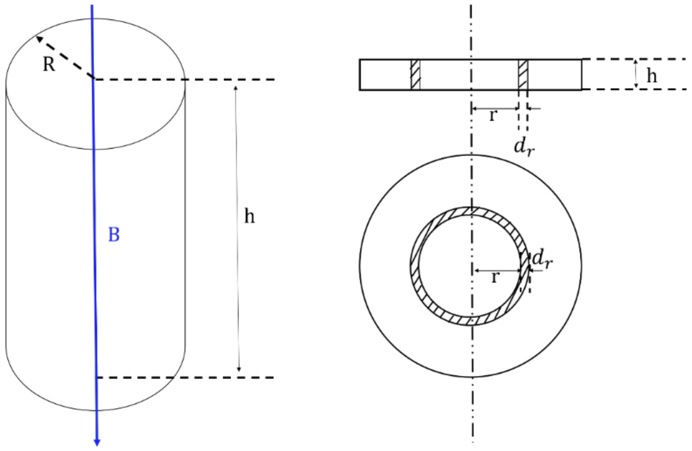

Take a small ring of the sample with $radius = r, width = d_r, height = h$ as indicated in the above figure. The resulting electromotive force $\varepsilon_{electrode}$ from the changing magnetic field is calculated as[2]

$$\varepsilon_{electrode} = -\oint \frac{dB}{dt} \cdot dS$$

Since magnetic field B is perpendicular to the cylinder, then we get[2]

$$\varepsilon_{electrode} = -\frac{dB}{dt}\int dS = -\frac{dB}{dt}\int 2\pi r dr = -\frac{dB}{dt}\pi r^2$$

In addition, the resistance of the ring is calculated as

$$dRES_{ring} = \rho\frac{2\pi r}{hdr}$$

where ρ is the material resistivity, $\rho_{GF} = 1\times10^{-3}\Omega\cdot m$, $\rho_{PtIr} = 2.4\times10^{-7}\Omega\cdot m$.[3,4]

According to Ohm's law, the current in the ring is calculated as

$$dI = -\frac{dB}{dt}\cdot\frac{h}{2\rho}rdr$$

Then the eddy current in the cylinder is calculated as[2]

$$I = \int dI = -\frac{dB}{dt}\cdot\frac{h}{2\rho}\int_0^R r\,dr = -\frac{1}{4\rho}\frac{dB}{dt}R^2h$$

And

$$(-\frac{dB}{dt})_{max} = SR_{max}\cdot X_{max}$$

Where SR_{max} =1170 T m^{-1} s^{-1} represents the maximum slew rate of the gradient, X_{max} = 15 cm is the maximum distance between the sample and the coil center[5].

Then we get

$$I_{max} = \frac{SR_{max} \cdot X_{max}}{4\rho} R^2 h$$

Then we get the calculated eddy currents

$$I_{\max\_GF} = 0.51\ \mu A$$

$$I_{\max\_PtIr} = 2.12\ mA$$

Decay time constant calculation. The induced eddy current *I* increases with *t* during linear ramp-up of the gradient as[4]

$$I(t) = I_{\max}(1 - e^{-\frac{t}{\tau}})$$

After reaching maximum, it decays as[4]

$$I(t) = I_{\max} \cdot e^{-\frac{t}{\tau}}$$

The decay time constant τ is calculated as[4]

$$\tau = \frac{L}{RES_{sample}}$$

Where the inductance *L* of the sample placed in the coil is calculated as[6,7]

$$L = \frac{\mu_0 h}{2\pi}\left(ln\frac{2h}{R} - 0.75\right)$$

Where μ_0 is the magnetic permeability of vacuum. $\mu_0 = 4\pi \times 10^{-7}\ H\ m^{-1}$.[2]

The resistance of the sample is calculated as

$$RES_{sample} = \frac{\varepsilon_{electrode}}{I_{max}}$$

Then we get the decay time constants

$$\tau_{GF} = 6.05\ ns$$

$$\tau_{PtIr} = 24200\ ns$$

We can see from the above results that the induced eddy currents for both electrodes are small and decay very fast compared with the repetition time and echo time used for our MRI acquisition: TE = 33 ms, TR = 2500 ms for T2 sequence, TE = 13 ms, TR = 500 ms for EPI sequence. Therefore, the influence of eddy current on artifact size is negligible.

Supplementary Figures

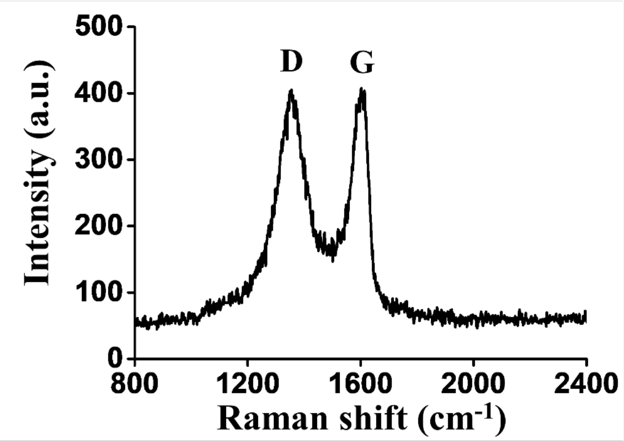

Supplementary Figure 1. Typical Raman spectrum from a dry GF under a 514-nm laser. Experiments were repeated three times with similar results. Source data are provided as a Source Data file.

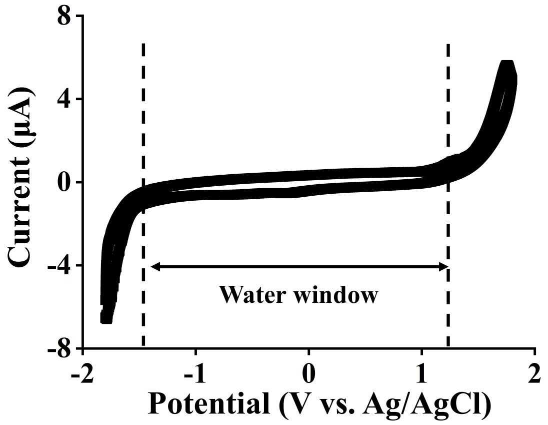

Supplementary Figure 2. Cyclic voltammogram of a GF electrodes measured between the voltage limits of -1.8 to 1.8 V (vs. Ag/AgCl electrode) for water window determination. The water oxidation and reduction potentials (water window) were determined as the potentials where steep increase in the current occurs. The water window of GFs, as indicated by the arrow, is from -1.5 to 1.3 V. Experiments were repeated three times with similar results. Source data are provided as a Source Data file.

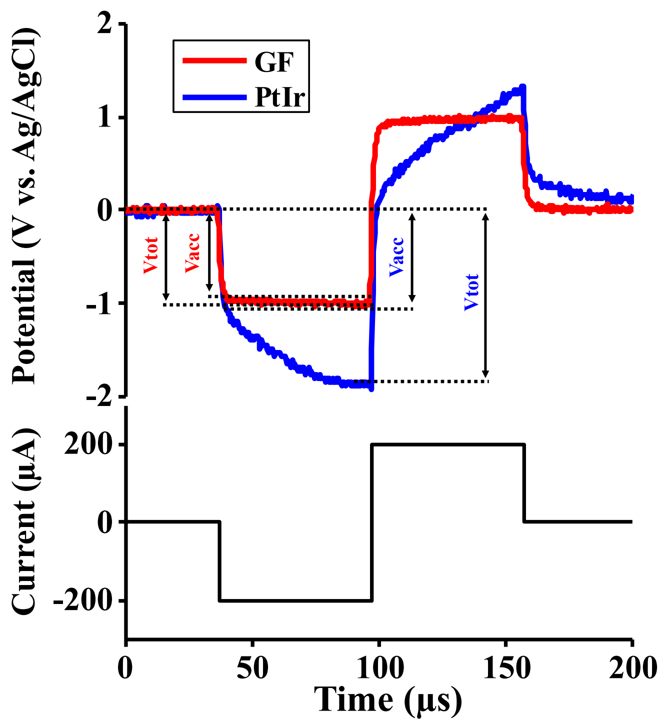

Supplementary Figure 3. Voltage transient of a GF (red) and PtIr (blue) electrode (upper curves) in response to a current pulse of 200 µA amplitude (lower curve). The pulse is biphasic and charge balanced with pulse duration of 60 µs and frequency of 130 Hz. V_{acc} is the access potential due to solution resistance. The negative potential excursion V_{exc} was calculated by subtracting the access potential V_{acc} from the total voltage V_{tot}. The charge injection limit was calculated by multiplying the current amplitude and pulse duration at which V_{exc} reaches the water reduction limit (-1.5 V and -0.6 V for GF and PtIr electrodes respectively), divided by the geometric surface area of the electrodes. V_{acc} and V_{tot} in red and blue are for GF and PtIr electrodes respectively. Source data are provided as a Source Data file.

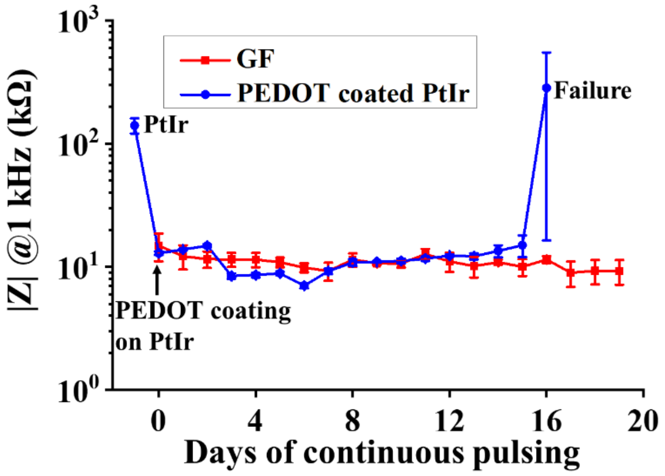

Supplementary Figure 4. Monitored impedance changes of GF and PEDOT modified PtIr electrodes under prolonged overpulsing. Overcurrent pulses with 1 mA amplitude were continuously applied. The PEDOT was electrochemically deposited onto the PtIr electrodes on day 0, as marked by the black arrow, which was accompanied by a significant decrease of the impedance. The impedance of the PtIr electrodes changed back to the values before PEDOT deposition on day 16, after 172.8 millions of cycles, indicating the pulse-induced degradation of the PEDOT coating. Distinctly, the GF electrodes show stable impedance values even after 19 d of continuous pulsing, indicating their high stability. The experiment was concluded after 19 d of continuous stimulation. Data represented as mean ± SD (n=5 electrodes). Source data are provided as a Source Data file.

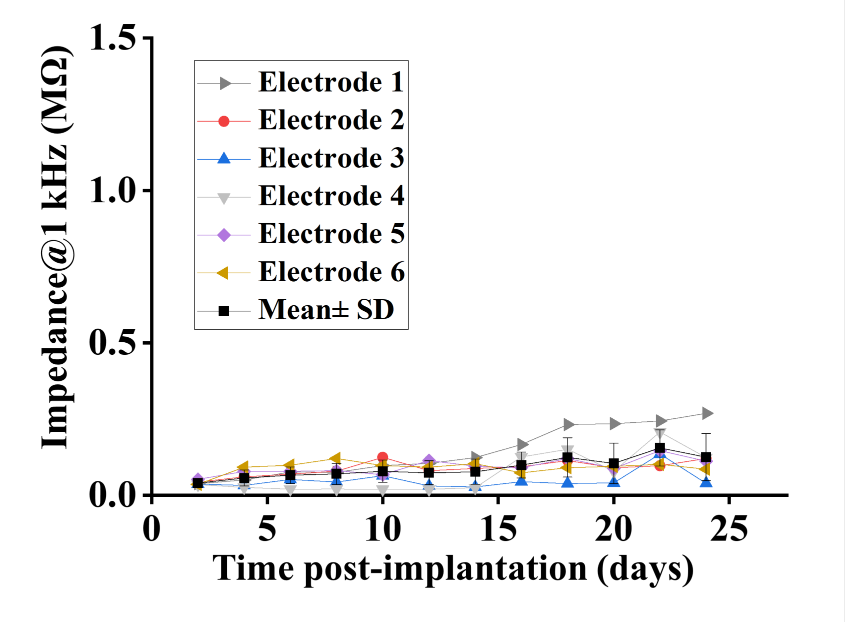

Supplementary Figure 5. Monitored impedance changes of GF electrodes chronically implanted in rat brains. It can be seen that the electrode impedances exhibited nearly constant values over time, indicating the stability of the GF electrodes *in vivo* in this tested timescale. n=6 electrodes. Source data are provided as a Source Data file.

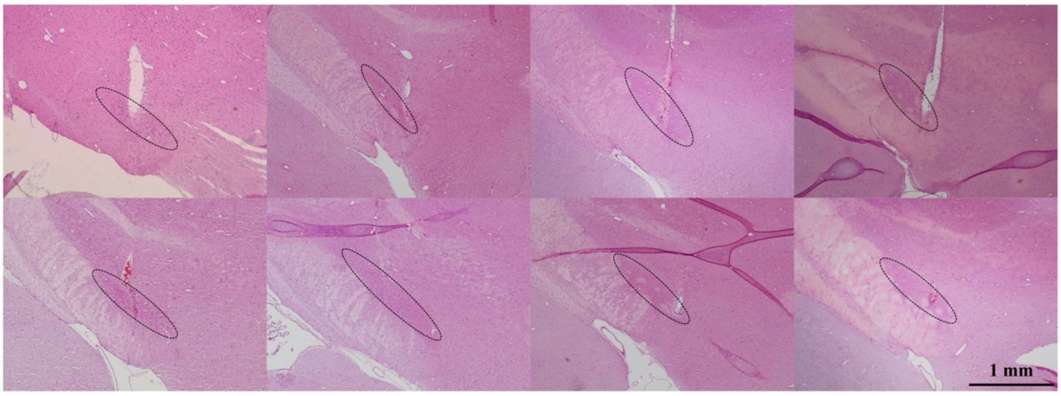

Supplementary Figure 6. Verification of the GF electrodes placement using H&E staining. The STN were marked with dotted ovals.

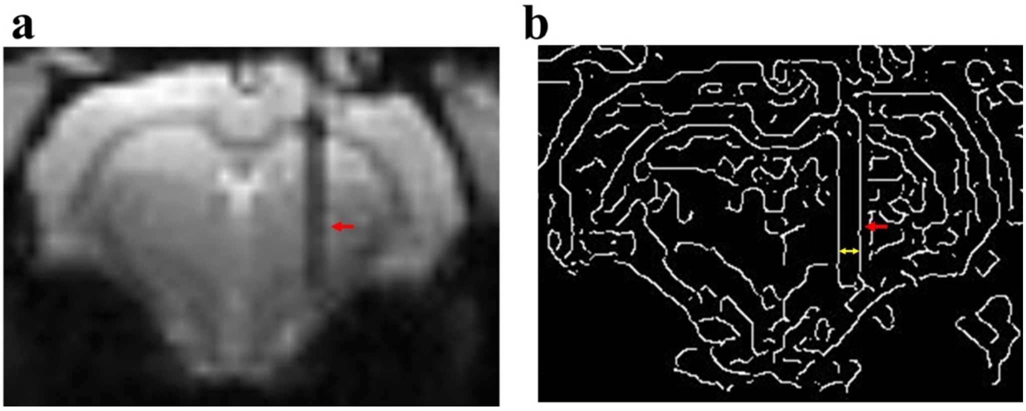

Supplementary Figure 7. An example showing the Canny edge detection used in the measurement of MRI artifact size. **a**, An original MRI image used for artifact measurement. **b**, The generated image after edge detection using Canny Edge Detector in Matlab (Mathworks, USA). The artifact and detected artifact edge are pointed by the red arrows. The size of the artifact (marked by the yellow arrow) is measured directly from the detected edge.

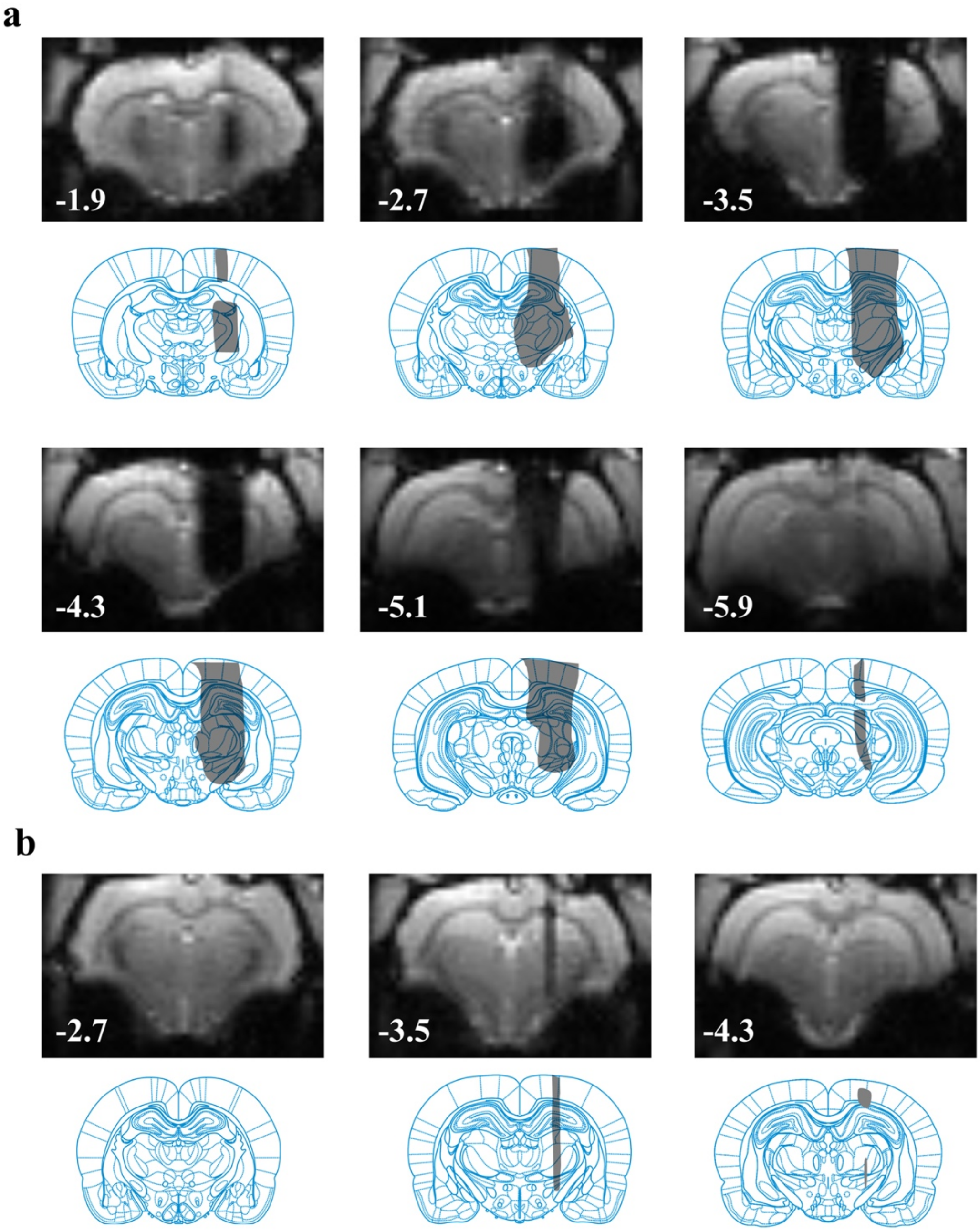

Supplementary Figure 8. EPI artifacts of GF and PtIr electrodes. Serial coronal scans (top rows) from rostral (left) to caudal (right) of EPI images from rat brains implanted with a PtIr (**a**) and GF (**b**) bipolar microelectrode. The outline of the artifact in each image is overlaid on the reference diagram of corresponding (closest) coronal section of the rat atlas (lower rows). The numbers in the images denote the relative distance from bregma (in mm). The schematic reference diagrams were adapted from the Paxinos and Watson (2014) [8].

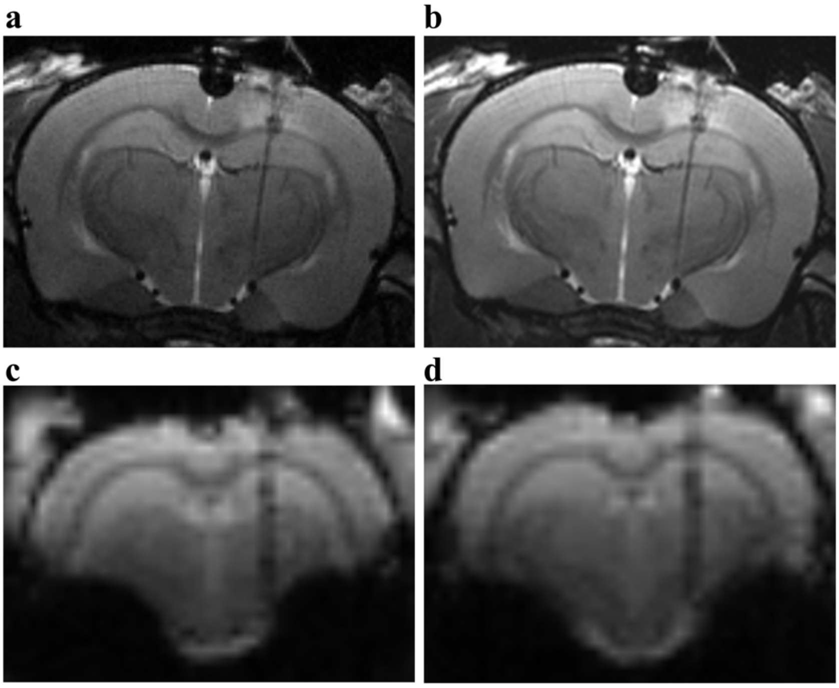

Supplementary Figure 9. Effect of DBS on MRI imaging. a, b, coronal sections of the T2-weighted images of a rat brain implanted with a GF bipolar microelectrode without (**a**) and with (**b**) DBS pulses. **c, d**, coronal sections of the EPI images of the rat brain implanted with a GF bipolar microelectrode without (**c**) and with (**d**) DBS pulses. The DBS pulses were same as what we used in the DBS-fMRI studies, with parameters as: 130 Hz, 300 μA, 60 μs duration, and biphasic. The results here indicate that the application of electrical stimulation pulses didn't induce additional artifact on the MRI scans.

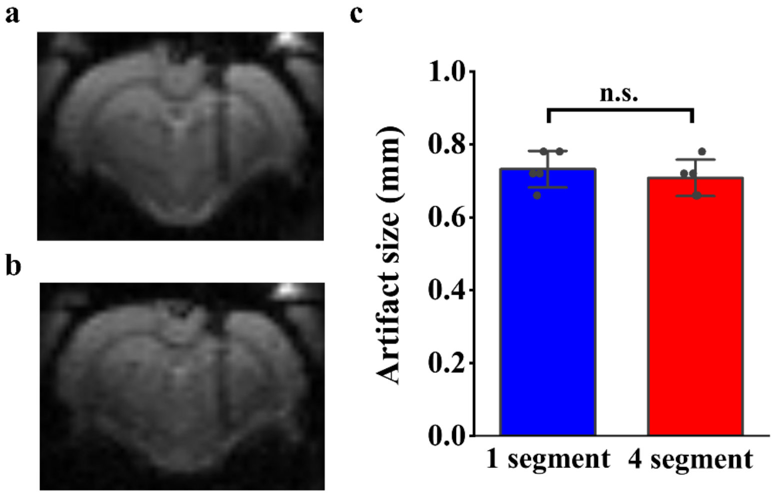

Supplementary Figure 10. EPI artifact size comparison of GF electrodes under one- and four-segment scans. a, b, Representative EPI images from rat brains implanted with a GF bipolar electrode under one- (**a**) and four-segment (**b**) scans. **c,** comparison of GF electrodes EPI artifact size measured from one- and four-segment scans. Data represented as mean ± SD (n = 5 electrodes, n.s.: not significant, two-tailed paired *t* test). Source data are provided as a Source Data file.

a

b

c

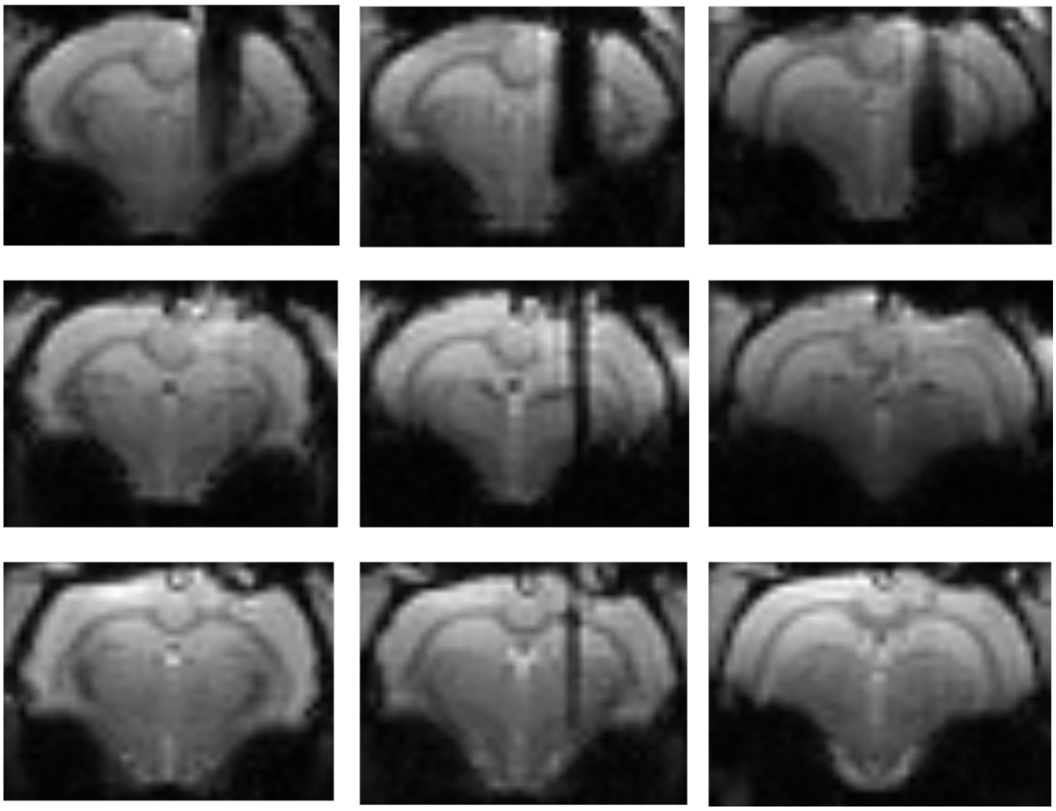

Supplementary Figure 11. Three serial coronal scans from rostral (left) to caudal (right) of EPI images from rat brains implanted with a tungsten (a), carbon fiber (CF, b) and GF (c) bipolar electrodes of same size. All electrodes are made of 75 μm diameter wires insulated with 5 μm thick Parylene-C. This gave final size of the bipolar electrodes in medial-lateral direction of 170 μm. 1K tow CFs which consists of approximately 1000 carbon filaments per tow (#CF701, The Composites Store, U.S.A.) was split to diameter of 75 μm and used to fabricate the CF electrodes here.

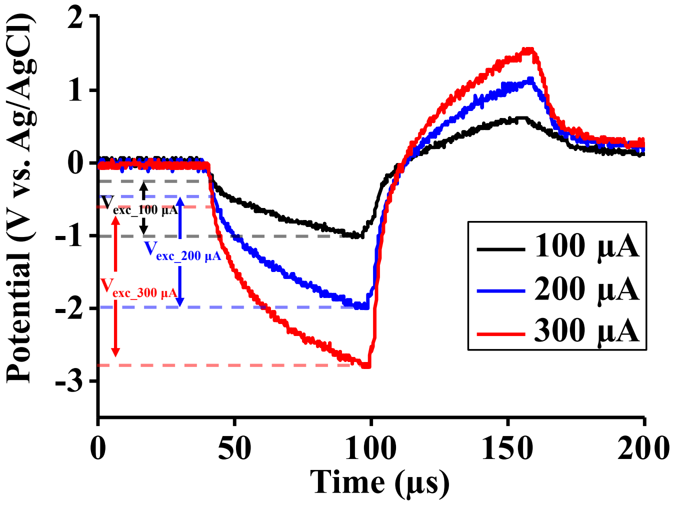

Supplementary Figure 12. Voltage transient of a 1K tow CF bipolar electrodes in response to a current pulse of different amplitude. The pulse is biphasic and charge balanced with pulse duration of 60 µs and frequency of 130 Hz (same as that in Supplementary Fig. 3). The negative potential excursion V_{exc} for each amplitude is marked. The V_{exc} at 200 µA amplitude reached the water reduction limit (-1.5 V). This result indicates that the CF can't go below 1 K tow in order to inject 200 µA current pulses without electrode potential being polarized beyond water window. The 1K tow CFs have a diameter of ~283 µm which gives a total size of ~586 µm (including 5 µm thick insulation layer) for the bipolar electrodes. Source data are provided as a Source Data file.

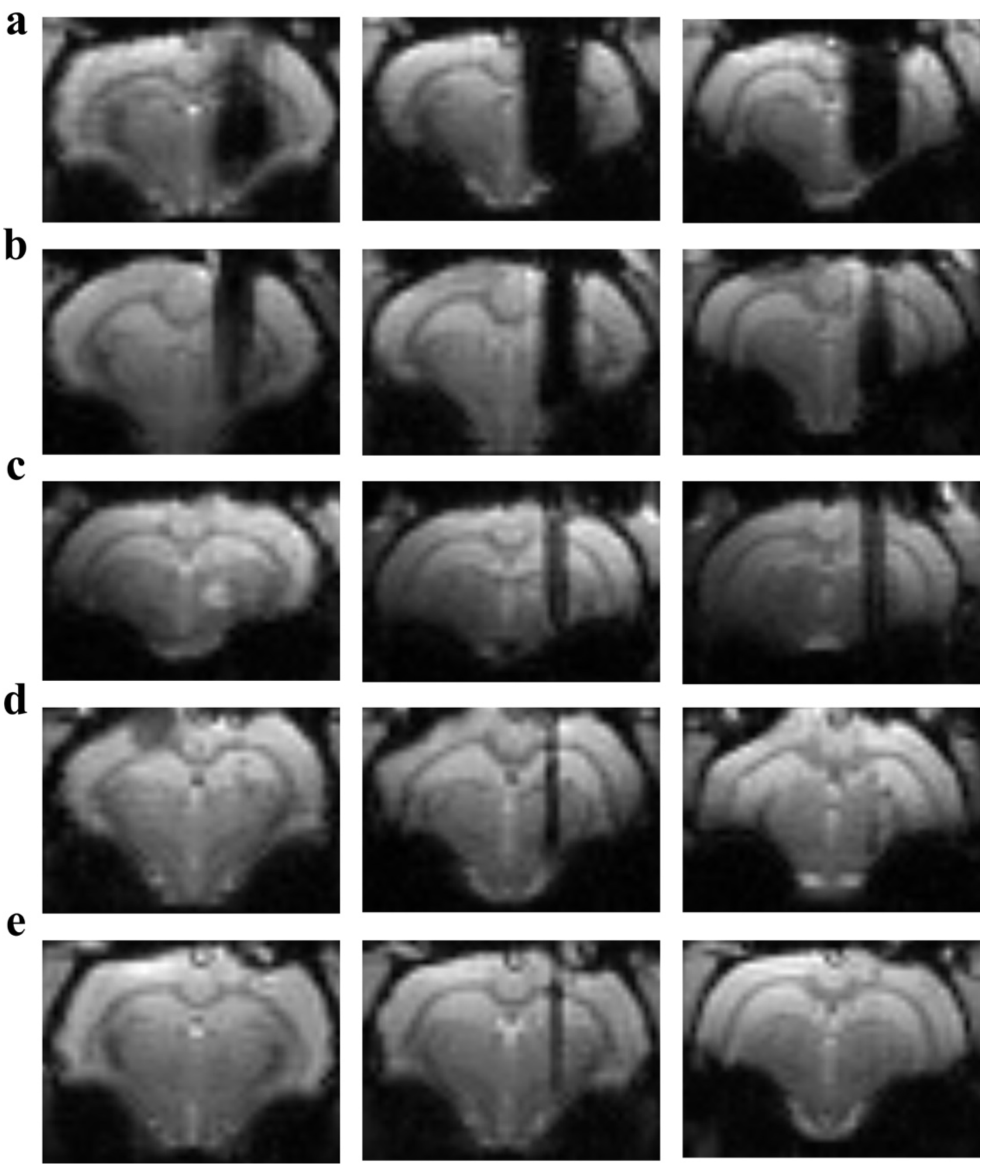

Supplementary Figure 13. Three serial coronal scans from rostral (left) to caudal (right) of EPI images from rat brains implanted with a PtIr (a), W (b), CF (c), G-Cu (d) and GF (e) bipolar electrodes. W, CF, and G-Cu bipolar electrodes are made from wires with minimum wire diameter d_{min} to inject 200 μA current pulses without polarizing the electrode potential beyond water window (see Supplementary Table 2 for details). For CFs, because single CFs with 175 μm diameter (d_{min} for CF) are not available on market, we used 1K tow CF (#CF701, The Composites Store, U.S.A.). The cross section of a 1K tow CF has same exposed area as that of a single CF with 175 μm diameter. PtIr and GF bipolar electrodes are made from PtIr and GF of 75 μm diameter.

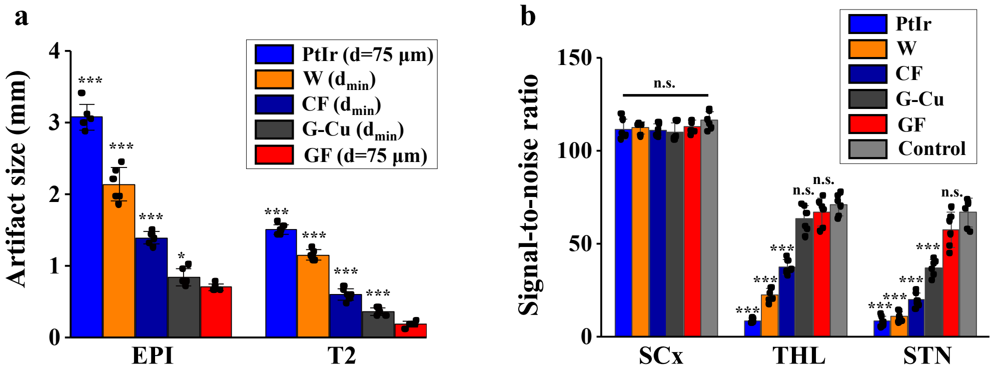

Supplementary Figure 14. MRI artifact sizes of various bipolar electrodes and corresponding SNR of the EPI signal in several ROIs. W, CF, and G-Cu bipolar electrodes are made from wires with minimum wire diameter d_{min} to inject 200 μA current pulses without polarizing the electrode potential beyond water window (same as those in Supplementary Fig. 13). PtIr and GF bipolar electrodes are made from PtIr and GF of 75 μm diameter. The ROIs and SNR of the EPI signal in control rats without any implant and rats implanted with various electrodes are defined and calculated as that in Fig. 3. **a**, Data represented as mean ± SD (n = 6 electrodes, * $p < 0.01$; ***$p < 0.001$, two-tailed unpaired t test). The statistical analysis is comparison with GF electrodes. **b**, Data represented as mean ± SD (n = 6 samples, ***$p < 0.001$; n.s.: not significant, two-tailed unpaired t test). The statistical analysis is comparison with control rats without any implant. Source data are provided as a Source Data file.

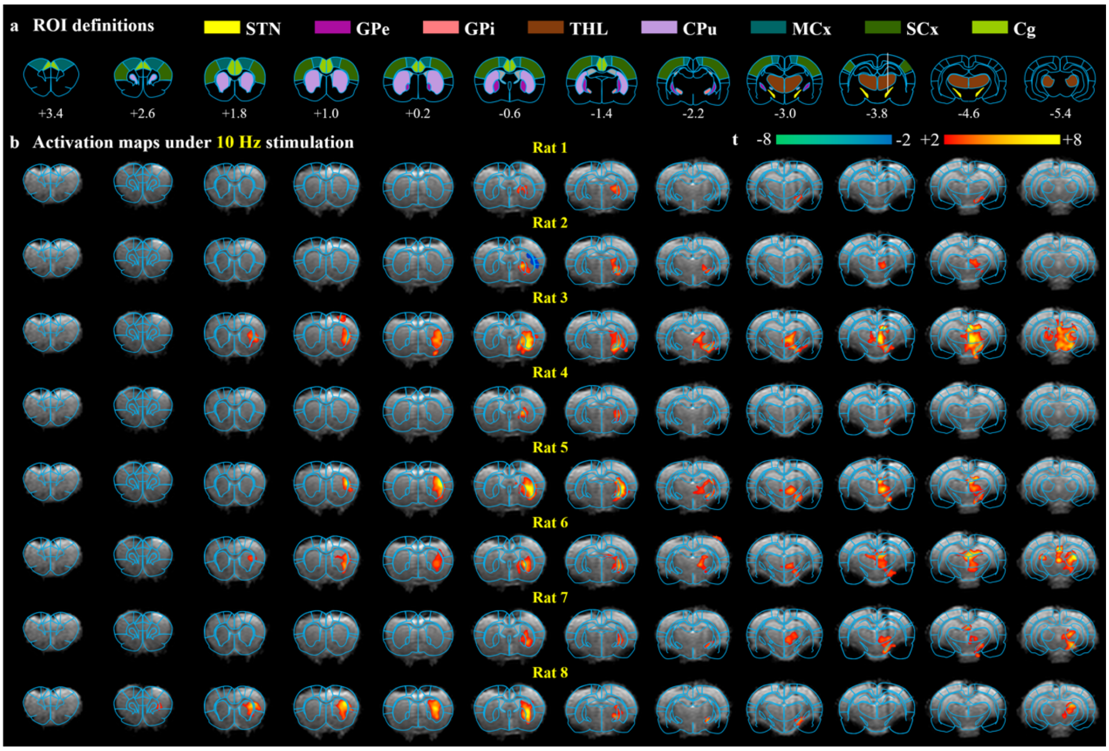
a ROI definitions
STN
GPe
GPi
THL
CPu
MCx
SCx
Cg
+3.4
+2.6
+1.8
+1.0
+0.2
-0.6
-1.4
-2.2
-3.0
-3.8
-4.6
-5.4
b Activation maps under 10 Hz stimulation
t -8 -2 +2 +8
Rat 1
Rat 2
Rat 3
Rat 4
Rat 5
Rat 6
Rat 7
Rat 8

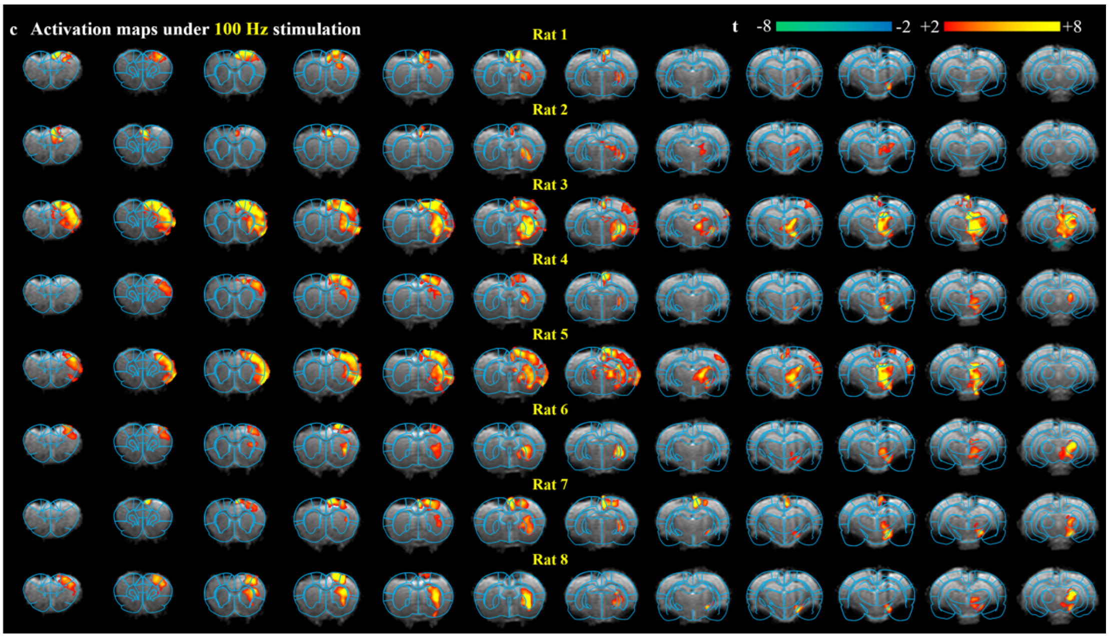
c Activation maps under 100 Hz stimulation
t -8 -2 +2 +8
Rat 1
Rat 2
Rat 3
Rat 4
Rat 5
Rat 6
Rat 7
Rat 8

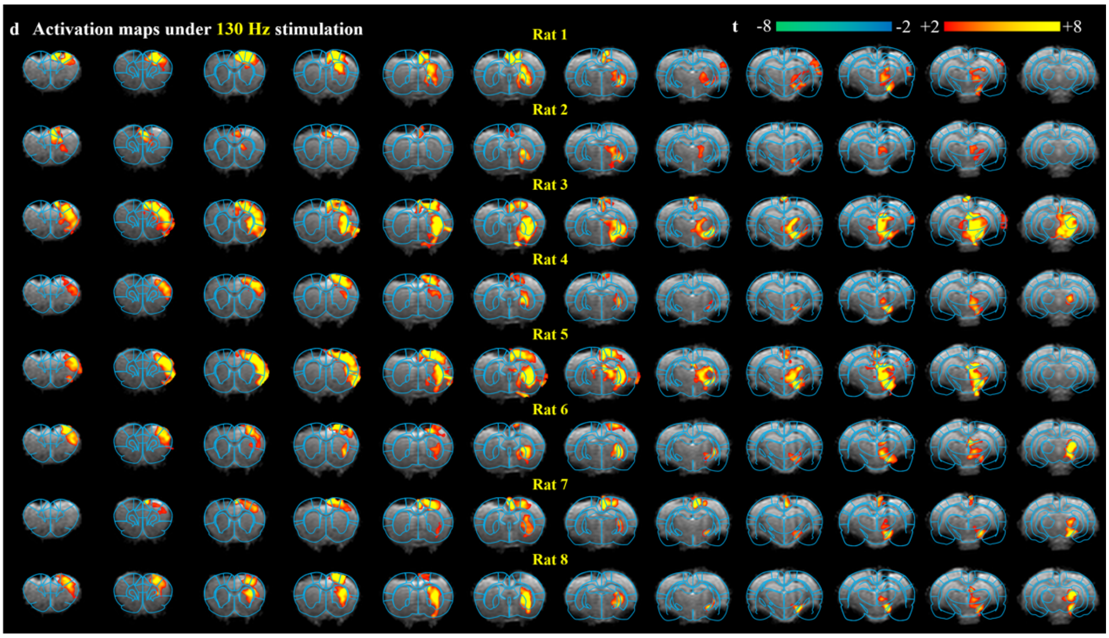

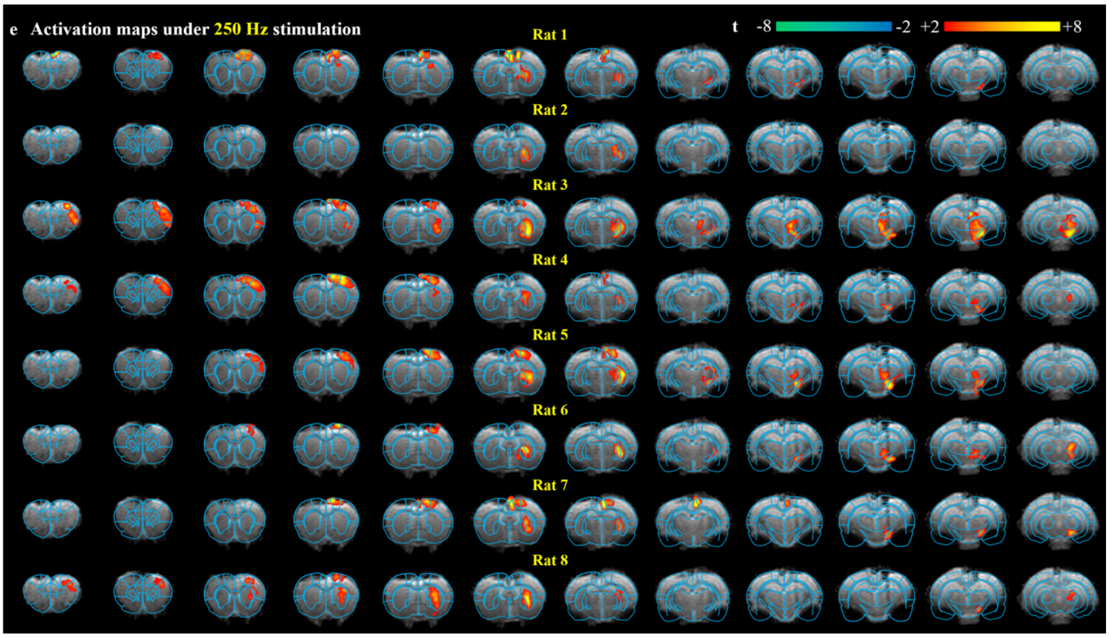

Supplementary Figure 15. BOLD activation maps from individual rats overlaid onto EPI images. Color bar denotes t-score values obtained by GLM analyses, with a significance threshold of uncorrected $p < 0.001$. n =3 scans from each rat.

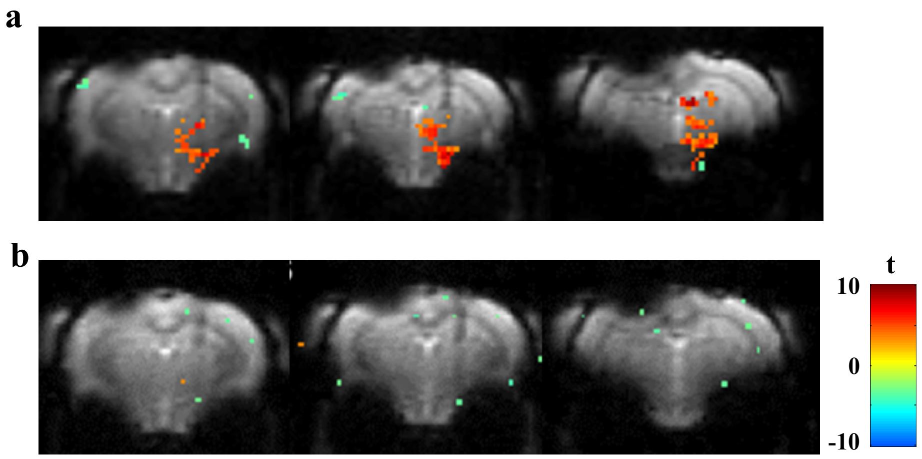

Supplementary Figure 16. BOLD activation maps evoked by STN-DBS with a GF electrode in a rat before (a) and after (b) sacrificing the animal. It can be seen that the sacrificed animal didn't show any BOLD response under same electrical pulses, indicating that the BOLD signals we observed in this study reflected brain activity rather than artifact from electrical pulses.

Supplementary Tables

PtIr					GF	Control
1.S1HL	25.CA1	49.V2ML	73.REth	97.A30	1.MPtA	1.MPtA
2.S1Tr	26.CA2	50.V1	74.PP	98.A29c	2.alv	2.alv
3.S1DZ	27.CA3	51.TG	75.LT	99.MPtA	3.Or	3.Or
4.M1	28.Rad	52.P1	76.ZIC	100.LPtA	4.Py	4.Py
5.Cg	29.SLu	53.LPMC	77.PPA	101.LPMR	5.Rad	5.Rad
6.LV	30.MoDG	54.bsc	78.SNL	102.LPLR	6.LMo1	6.LMo1
7.st	31.LMo1	55.DLG	79.SNCD	103.DLG	7.MoDG	7.MoDG
8.cst	32.GrDG	56.ILG	80.SNR	104.PrG	8.PoDG	8.PoDG
9.RtSt	33.PoDG	57.APTD	81.fmj	105.Po	9.CA1	9.CA1
10.LVDL	34.EP	58.PLi	82.dhc	106.OPC	10.CA3	10.CA3
11.AVVL	35.B	59.APTV	83.DS	107.opt	11.LPMP	11.LPMP
12.EGP	36.LDDM	60.SG	84.Zo	108.sox	12.LPLR	12.LPLR
13.ic	37.LDVL	61.MGD	85.SuG	109.cp	13.Po	13.Po
14.Rt	38.VPL	62.MGM	86.Op	110.STN	14.VPM	14.VPM
15.VL	39.VPM	63.MGV	87.InG	111.ZIV	15.ZID	15.ZID
16.alv	40.VM	64.MZMG	88.InWh	112.ZID	16.ZIV	16.ZIV
17.Or	41.ZI	65.PIL	89.DpG	113.ns	17.STN	17.STN
18.Py	42.Ang	66.PP	90.APT	114.scp	18.m1	18.m1
19.ml	43.PC	67.str	91.PoT	115.LHbL		
20.SPFPC	44.CL	68.IGL	92.SNL	116.V2MM		
21.VPPC	45.MDL	69.PrG	93.fi	117.V2		
22.PaF	46.MDC	70.DLG	94.Or	118.F		
23.fr	47.ns	71.IMA	95.SubG			
24.PR	48.mfb	72.LRLR	96.PtPD			

Supplementary Table 1. Obstructed brain regions by EPI artifacts of GF and PtIr bipolar electrodes. All brain nuclei overlapping with the artifacts are numbered and listed based on The Rat Brain Atlas of Paxinos & Watson[8], although some of them are not completely obstructed. For GF electrodes, no brain nuclei are completely obstructed. The brain nuclei listed in the column under “Control” are those passed through by the real electrode tracks. It can be seen that no additional nuclei except for those passed through by the real electrode track was affected by the GF electrode artifact, indicating that the GF electrodes have minimal artifact.

	CIL (mC cm^{-2})	d_{min} (μm)
Tungsten	0.28	72
CF	0.05	175*
PtIr	0.15	100
G-Cu	0.057	175
GF	10.1	12

Supplementary Table 2. Measured CIL and calculated minimum wire diameter d_{min} for electrodes to inject 200 μA current pulses (60 μs duration) without polarizing the electrode potential beyond water window. Current pulse parameters were as same as those in Supplementary Fig. 3. The measurement of CIL were as described in Methods and Supplementary Fig. 3. Water reduction limit of -1.5 V was used for all electrodes except PtIr electrodes which used -0.6 V threshold. For each material, minimum wire diameter d_{min} was calculated as:

$$\pi \cdot (\frac{d_{min}}{2})^2 = \frac{current\ amplitude \times duration}{CIL}$$

Where current amplitude is 200 μA and duration is 60 μs.

*175 μm is the calculated diameter for single CFs. Single CFs of 175 μm diameter is not available on market, we used 1K tow CFs which has same cross-sectional area with that of single CFs with 175 μm diameter for artifact assessment in Supplementary Fig. 13 and 14.

Supplementary References:

1 Lu, L. et al. Soft and MRI Compatible Neural Electrodes from Carbon Nanotube Fibers. *Nano Lett*. **19**, 1577-1586 (2019).

2 Xie, B. Chen, Y. College Physics Tutorials Vol.2 (Wuhan University of Technology Press, Wuhan, 2014)

3 Zelin, D. et al. Facile Fabrication of Light, Flexible and Multifunctional Graphene Fibers. *Adv. Mater*. **24**, 1856-1861 (2012)

4 Jiang, C. Q., Hao, H. W. & Li, L. M. Artifact properties of carbon nanotube yarn electrode in magnetic resonance imaging. *J. Neural Eng*. **10**, 026013 (2013).

5 Manual book of 9.4 T MR scanner, Bruker, German.

6 Xu, B. Practical Electrical Calculation Manual, 3rd edition (Shanghai Scientific & Technical Publishers, Shanghai, 2008)

7 Rodriguez, J. J. Impedance spectroscopy for characterization of biological matter (Logos Verlag Berlin GmbH, Berlin, 2018).

8 Paxinos, G. and Watson, C. The Rat Brain in Stereotaxic Coordinates - Seventh Edition. (Academic Press, London, 2014).
