# Supplementary material for: Full activation pattern mapping by simultaneous deep brain stimulation and fMRI with graphene fiber electrodes
Source: Nat Commun. 2020 Apr 14;11:1788. doi: 10.1038/s41467-020-15570-9 (PMC7156737; doi:10.1038/s41467-020-15570-9)
Supplement: Supplementary file 2 — Reporting Summary [file 41467_2020_15570_MOESM2_ESM.pdf]

nature research

Corresponding author(s): Xiaojie Duan

Last updated by author(s): Feb 9, 2020

Reporting Summary

Nature Research wishes to improve the reproducibility of the work that we publish. This form provides structure for consistency and transparency in reporting. For further information on Nature Research policies, see Authors & Referees and the Editorial Policy Checklist .

Statistics

For all statistical analyses, confirm that the following items are present in the figure legend, table legend, main text, or Methods section.

n/a	Confirmed	
☐	☒	The exact sample size (*n*) for each experimental group/condition, given as a discrete number and unit of measurement
☐	☒	A statement on whether measurements were taken from distinct samples or whether the same sample was measured repeatedly
☐	☒	The statistical test(s) used AND whether they are one- or two-sided<br>*Only common tests should be described solely by name; describe more complex techniques in the Methods section.*
☐	☒	A description of all covariates tested
☐	☒	A description of any assumptions or corrections, such as tests of normality and adjustment for multiple comparisons
☐	☒	A full description of the statistical parameters including central tendency (e.g. means) or other basic estimates (e.g. regression coefficient) AND variation (e.g. standard deviation) or associated estimates of uncertainty (e.g. confidence intervals)
☐	☒	For null hypothesis testing, the test statistic (e.g. *F*, *t*, *r*) with confidence intervals, effect sizes, degrees of freedom and *P* value noted<br>*Give P values as exact values whenever suitable.*
☒	☐	For Bayesian analysis, information on the choice of priors and Markov chain Monte Carlo settings
☐	☒	For hierarchical and complex designs, identification of the appropriate level for tests and full reporting of outcomes
☐	☒	Estimates of effect sizes (e.g. Cohen's *d*, Pearson's *r*), indicating how they were calculated

Our web collection on statistics for biologists contains articles on many of the points above.

Software and code

Policy information about availability of computer code

Data collection	Hitachi S4800 Scanning Electron Microscope software for SEM image collection;LabSpec Version 5.36.11 for Raman data collection;CHI660E Electrochemical Workstation Version 15.08 for electrochemical data collection; ANY-maze Version 4.70 software for open field test data collection;ParaVision Version 6.0.1 for MRI acquisitions;
Data analysis	ANY-maze Version 4.70 software for open field test data analysis;Origin 2020 for plotting data and data analysis; MATLAB R2018a toolbox, spm12 for fMRI data analysis; Canny Edge Detector in Matlab R2018a for artifact edge.

For manuscripts utilizing custom algorithms or software that are central to the research but not yet described in published literature, software must be made available to editors/reviewers. We strongly encourage code deposition in a community repository (e.g. GitHub). See the Nature Research guidelines for submitting code & software for further information.

Data

Policy information about availability of data

All manuscripts must include a data availability statement. This statement should provide the following information, where applicable:
- Accession codes, unique identifiers, or web links for publicly available datasets
- A list of figures that have associated raw data
- A description of any restrictions on data availability

The authors declare that the data supporting the findings of this study are available within the article and its Supplementary Information files or available from the corresponding authors upon reasonable request, including all raw MRI image files. The source data underlying Figs 1e-i, 2b-g, 3g-h and 5a-h and Supplementary Figs 1, 2, 3, 4, 5, 9c, 11, 13a-b and 16b are provided as a Source Data file.

Field-specific reporting

Please select the one below that is the best fit for your research. If you are not sure, read the appropriate sections before making your selection.

- [x] Life sciences
- [ ] Behavioural & social sciences
- [ ] Ecological, evolutionary & environmental sciences

For a reference copy of the document with all sections, see nature.com/documents/nr-reporting-summary-flat.pdf

Life sciences study design

All studies must disclose on these points even when the disclosure is negative.

Sample size	Sample sizes were estimated based on previous similar studies. [Lee, J. H. et al. Global and local fMRI signals driven by neurons defined optogenetically by type and wiring. Nature 465, 788 (2010).]
Data exclusions	Methods, 'Animal surgery'subsection, paragraph 1&2. The exclusion criteria were pre-established.<br>The contralateral rotational number was counted for 5 minutes and those exhibiting a contralateral rotation speed exceeding 15 turns/min were considered as successful and selected for electrode implantation.<br>Electrode tip placements within the STN were verified for each subject by T2-weighted RARE anatomical MRI images acquired immediately after implantation and H&E staining of the coronal brain sections at the end of the study. Animals with electrode placements outside of the target regions were discarded from the study and excluded from all further experimental analyses.
Replication	The number of repetitions for each experiment has been indicated in the manuscript.
Randomization	The animals were randomizedly chosen for treatment to ensure reliance of each experiment.
Blinding	The animals were randomizedly chosen for treatment to ensure reliance of each experiment.

Reporting for specific materials, systems and methods

We require information from authors about some types of materials, experimental systems and methods used in many studies. Here, indicate whether each material, system or method listed is relevant to your study. If you are not sure if a list item applies to your research, read the appropriate section before selecting a response.

Materials & experimental systems

n/a	Involved in the study	
x		Antibodies
x		Eukaryotic cell lines
x		Palaeontology
	x	Animals and other organisms
x		Human research participants
x		Clinical data

Methods

n/a	Involved in the study	
x		ChIP-seq
x		Flow cytometry
	x	MRI-based neuroimaging

Animals and other organisms

Policy information about studies involving animals; ARRIVE guidelines recommended for reporting animal research

Laboratory animals	Adult male Sprague-Dawley rats weighing 250-280 g and 8-10 weeks of age (Charles River Laboratories, China) were used throughout this study.
Wild animals	The study did not involve wild animals.
Field-collected samples	The study did not involve samples collected from the field.
Ethics oversight	Our procedures for handling the animals complied with the Beijing Administration Rules of Laboratory Animals and the National Standards of Laboratory Animal Requirements of Environment and Housing Facilities (GB 14925–2010) and were approved by the Institutional Animal Care and Use Committee of Peking University.

Note that full information on the approval of the study protocol must also be provided in the manuscript.

Magnetic resonance imaging

Experimental design

Design type	Task state; Block design.
Design specifications	The number of blocks is 1 in each per session, it starts the onsets of task at 60 s and continued for 30 s.
Behavioral performance measures	No behavioral measures were made during fMRI as the animals were anesthetized.

Acquisition

Imaging type(s)	Functional; Structural.
Field strength	9.4T
Sequence & imaging parameters	T2-weighted anatomical images were acquired using RARE sequence (TR/TE= 2500/33 ms, FOV= 3 × 3 cm, slice thickness = 0.8 mm, matrix = 256 × 256). fMRI scans using 4-shot gradient echo EPI sequence (TR/TE =500/13 ms, FOV = 3 × 3 cm, slice thickness = 0.8 mm, matrix =80 × 80).
Area of acquisition	Whole brain

Diffusion MRI ☐ Used ☒ Not used

Preprocessing

Preprocessing software	MATLAB R2017a with the toolbox spm12.EPI images were realigned first for motion correction, and coregistered to the anatomical template, and spatially smoothed at full-width at half maximum (FWHM) of 0.8 × 0.8 mm.
Normalization	The fmri data was coregistered to the subject's own T2 anatomical images, which were further coregistered to a rat brain template using a normalized mutual information metric, both steps being affine transformation.
Normalization template	The in vivo MRI template of Valdés-Hernández et al. (2011).
Noise and artifact removal	Motion parameters were used as regressor in first level general linear model.
Volume censoring	Anaesthetized rats yielded low level motions (maximum translation distance < 0.01 mm, maximum rotation degree < 0.5°), estimated by the realignment parameters from SPM12. Therefore, no fMRI volume was censored during the preprocessing.

Statistical modeling & inference

Model type and settings	Data were conducted across subjects using general linear modeling with reference to the stimulation paradigm.
Effect(s) tested	Deep brain stimulation

Specify type of analysis: ☐ Whole brain ☒ ROI-based ☐ Both

Anatomical location(s): Regions of interest (ROIs) were defined on an MRI atlas (Valdés-Hernández et al. 2011).

Statistic type for inference (See Eklund et al. 2016)	One sample T test with significance level at p < 0.001.
Correction	One sample T test with a significance threshold of uncorrected p < 0.001. [Jung, W. B., Shim, H.-J. & Kim, S.-G. Mouse BOLD fMRI at ultrahigh field detects somatosensory networks including thalamic nuclei. Neuroimage 195, 203-214 (2019).]

Models & analysis

n/a	Involved in the study
☒	☐ Functional and/or effective connectivity
☒	☐ Graph analysis
☒	☐ Multivariate modeling or predictive analysis
